# Synthesis of Flavonols and Assessment of Their Biological Activity as Anticancer Agents

**DOI:** 10.3390/molecules29092041

**Published:** 2024-04-28

**Authors:** Yu-Hui Hsieh, Pei-Hsuan Hsu, Anren Hu, Yang-Je Cheng, Tzenge-Lien Shih, Jih-Jung Chen

**Affiliations:** 1 Biomedical Industry Ph.D. Program, School of Life Sciences, National Yang Ming Chiao Tung University, Taipei 112304, Taiwan; hsieh.ls10@nycu.edu.tw; 2 Department of Chemistry, Tamkang University, New Taipei City 251301, Taiwan; peggy060708@gmail.com (P.-H.H.); jason0110422@gmail.com (Y.-J.C.); 3 Department of Laboratory Medicine and Biotechnology, Tzu Chi University, Hualien 970374, Taiwan; anren@mail.tcu.edu.tw; 4 Department of Pharmacy, School of Pharmaceutical Sciences, National Yang Ming Chiao Tung University, Taipei 112304, Taiwan; 5 Department of Medical Research, China Medical University Hospital, China Medical University, Taichung 404333, Taiwan; 6 Traditional Herbal Medicine Research Center, Taipei Medical University Hospital, Taipei 110301, Taiwan

**Keywords:** anticancer agent, human non-small cell lung cancer cell, flavonol

## Abstract

A series of flavanols were synthesized to assess their biological activity against human non-small cell lung cancer cells (A549). Among the sixteen synthesized compounds, it was observed that compounds **6k** (3.14 ± 0.29 µM) and **6l** (0.46 ± 0.02 µM) exhibited higher potency compared to 5-fluorouracil (5-Fu, 4.98 ± 0.41 µM), a clinical anticancer drug which was used as a positive control. Moreover, compound 6l (4’-bromoflavonol) markedly induced apoptosis of A549 cells through the mitochondrial- and caspase-3-dependent pathways. Consequently, compound **6l** might be developed as a candidate for treating or preventing lung cancer.

## 1. Introduction

**Figure 1:**
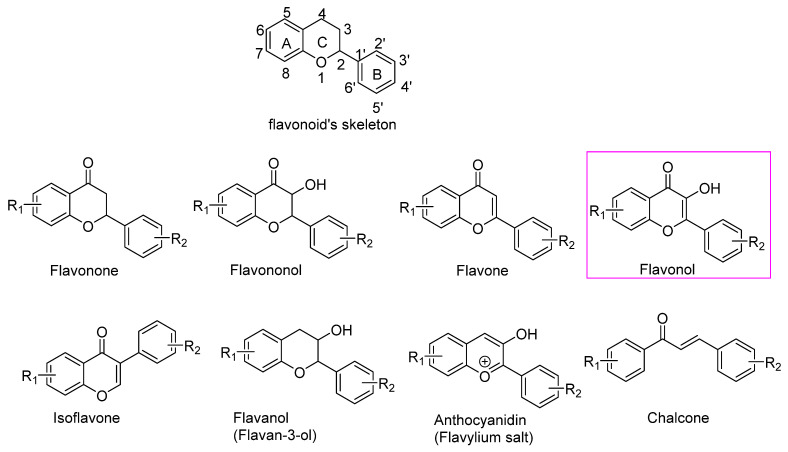
Flavonoid’s skeleton and its subfamily members.

**Figure 2:**
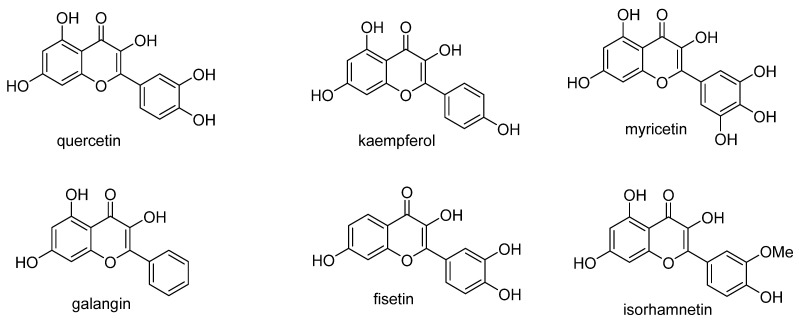
The representative structures of flavonols.

Flavonoids are naturally occurring polyphenolic compounds. More than 10,000 flavonoids have been detected and categorized into subclasses [1]. They are isolated from a wide range of plant families and species, and exhibit certain pharmacological activities such as antioxidant [2], anti-inflammatory [3,4], antimicrobial [5,6], antiallergenic [7], anticancer [8,9] and antiviral [10]. Flavonoids are natural antioxidants since they possess a reactive oxygen species (ROS) that will damage the membranes and DNA in mammals [11]. The various classes of flavonoids differ in the level of oxidation and pattern of substitution on the C ring (Figure 1). The double bond between C2-C3 and the oxo group at C4 of C ring, and the position of the B ring are crucial determinants for their anticancer activity [12]. Flavonoids act by multiple mechanisms but further studies on target selectivity and specificity of flavonoids are necessary to establish them as anticancer therapeutics [12]. The most studied flavonols, a class of flavonoids, are quercetin, kaempferol, galangin, and myricetin, widely present in fruits, vegetables, tea, cocoa, and red wine (Figure 2) [13]. In addition, previous research results indicate the inhibitory effects of flavonoids such as apigenin and luteolin as well as the flavonol quercetin and its derivatives on various leukemia cell lines [14]. These natural compounds can be prototypes for broad-spectrum chemotherapy drugs [14]. Flavonoids have been reported to have an excellent safety profile (no toxicity at up to 140 g/day), with no known significant adverse effects [15]. Pietta et al. reported that the 3-OH group in the C ring is essential to generate a high radical-scavenging activity [16]. Additionally, antioxidants help human beings reduce cancer risks [17]. Furthermore, this activity is enhanced when an additional hydroxyl group, such as myricetin, is present on the B ring [16]. Research reports that quercetin (Figure 2) has multiple pharmacological properties, including neuroprotective [18], anticancer [19,20,21,22], and antiviral [23,24] properties.

Natural and synthetic flavonoids have been developed as agents against non-small lung cancer [19,25]. Previously, we reported the synthesis of halo-substituted chalcones and azachalcones to inhibit the pro-inflammatory response [26]. Since flavonols can be synthesized from chalcones, we aim to explore the potential of halo-substituted flavonols. Therefore, in this study, we investigated the activity of sixteen synthesized flavonols against human non-small lung cancer cells (A549).

## 2. Results and Discussion

### 2.1. Chemistry

**Scheme 1:**
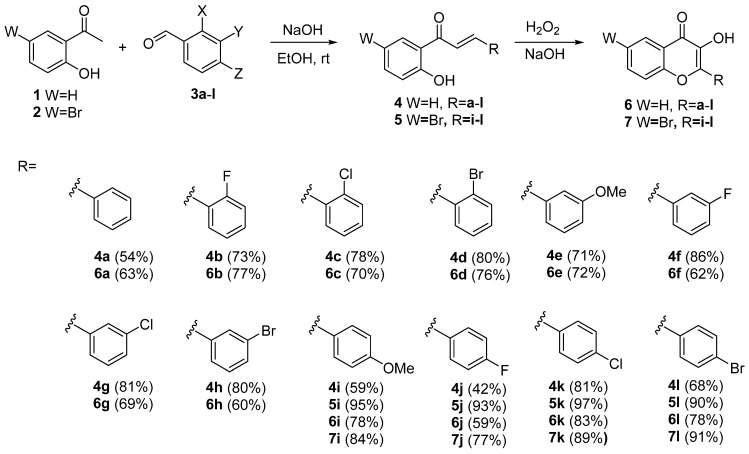
Synthesis of flavonols.

The synthetic strategy for the target molecules is presented in Scheme 1. The most common method for synthesizing chalcones is Claisen–Schmidt’s condensation [27]. Chalcone is one of the precursors in the biosynthesis of flavonoids. The reaction of 2’-hydroxyacetophenone (**1**) and 5-bromo-2-hydroxyacetophenone (**2**) with the corresponding aldehydes (**3a**–**l**) under NaOH/EtOH condition afforded chalcones **4a**–**l** (42–81%) and **5i**–**l** (90–97%), respectively, which were then subjected to the Algar–Flynn–Oyamada reaction (H_2_O_2_/NaOH) [28,29] and afforded the target flavonols **6a**–**l** (59–83%) and **7i**–**l** (77–91%), respectively. The synthesis of compounds **4**–**7** is facile, and their purification involves simple filtration, followed by washing with ethanol or methanol to obtain pure target molecules.

### 2.2. Pharmacology/Biology

The sixteen synthesized compounds were evaluated for their inhibitory activities against the growth of human non-small cell lung cancer A549 cells. The results revealed that compound **6l** (with a 4’-bromo substitution) exhibited the most potent inhibitory activity against the A549 cells (IC_50_ = 0.46 ± 0.02 μM), which was much better than that of the positive control, 5-fluorouracil (5-FU) (IC_50_ = 4.98 ± 0.41 μM) (Table 1). Compound **6k** (with a 4’-chloro substitution, IC_50_ = 3.14 ± 0.29 μM) also showed better inhibitory activity than 5-FU. Moreover, compounds **6a** (without substitution) and **6j** (with a 4’-fluoro substitution) also exhibited effective inhibitions with IC_50_ values of 6.34 ± 0.89 and 6.13 ± 0.63 μM, respectively. Consequently, a halogen at C-4’ in the B ring may induce cytotoxic effects against A549 cells [30,31].

**Figure 3:**
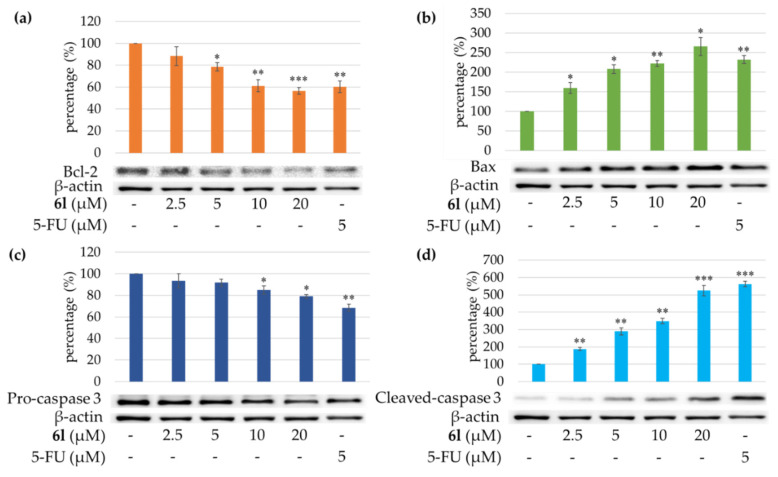
Western blot analysis for Bcl-2 (**a**), Bax (**b**), pro-caspase-3 (**c**), and cleaved caspase-3 (**d**) in each group on A549 cells. **6l** means compound **6l**. 5-Flurouracil (5-FU) was used as a positive control. Asterisks indicate significant differences (* *p* < 0.05, ** *p* < 0.01, and *** *p* < 0.001) compared with the control group.

The effect of treating the A549 cells with compound 6l (20 μM) on the expression of apoptosis-related proteins was investigated (Figure 3). The results revealed a decrease in the expression level of the anti-apoptotic protein Bcl-2, while that of the pro-apoptotic protein Bax increased. Caspase-3 activation is a hallmark of apoptosis. Thus, compound **6l** increases the expression level of cleaved caspase-3 (active caspase-3). The results showed that compound **6l** induced apoptosis in A549 cells through mitochondrial- and caspase-3-dependant pathways.

## 3. Experimental

### 3.1. General Procedures

All chemicals were purchased from either Acros or Alfa from Uniworld in Taiwan. The ^1^H- and C^13^-NMR data were recorded on a Bruker 600 MHz Ultrashield instrument (Bruker, Billerica, MA, USA). The chemicals were reported in parts per million (ppm) relative to the residual solvent (^1^H for DMSO-*d*_6_: 2.49 ppm; ^13^C NMR for DMSO-*d*_6_: 39.7 ppm). The reaction progress was monitored by thin-layer chromatography (Analtech Silica gel HLF UV254, Analtech Inc. Newark, DE, USA) and stained with either KMnO_4_ or p-anisaldehyde solutions. The melting points were determined by an open capillary tube on an MP-2 apparatus. The molecular weights of compounds were determined by a Thermo LCQ Fleet ion trap mass spectrometer.

### 3.2. Mass Determination

Using a micropipette, the sample (1 μL) was loaded onto a graphite paper cut into a triangle (10 × 10 mm), washed with MeOH, and cleaned with Kimwipe paper. An ion trap mass spectrometer (LCQ Duo, Finnigan, San Jose, CA, USA) equipped with a paper-spray ionization (PSI) source was employed to determine the molecular weights of the samples. A high voltage (3.5 kV) was applied for sample ionization. The MS spectra scans were collected in the positive and negative ion modes in the *m/z* range of 100–400.

### 3.3. Chemistry

#### 3.3.1. General Preparation of Chalcones

NaOH (50%, 3.0 equiv.) was added to a solution of acetophenone (1.0 equiv.) in EtOH (0.2 M) and stirred at ambient temperature for 30 min. Subsequently, the corresponding aldehyde (1.2 equiv.) was added to the mixture in EtOH (0.2 M) at ambient temperature. The progress of the reaction was monitored by thin-layer chromatography (TLC) until the aldehyde was consumed. The mixture was acidified with HCl (2N) and added distilled water. The precipitate was filtered by suction filtration and washed with EtOH to obtain chalcones **4a**–**l** and **5i**–**l**.

#### 3.3.2. General Preparation of Flavonols

A solution of the corresponding 2-hydroxyacetophenone (1.0 equiv.) and NaOH (50%, 5.0 equiv.) in MeOH was added to H_2_O_2_ (35%). The mixture was stirred in an ice bath and TLC monitored the progress of the reaction. At the end of the reaction, the mixture was acidified with HCl (2N), and distilled water was added to allow a precipitate formation. The precipitate was washed with cold MeOH to obtain flavonols **6i**–**l** and **7i**–**l**.

#### 3.3.3. 3-Hydroxy-2-phenyl-4H-chromen-4-one (**6a**)

Yield: 63% (75% [32]). A yellow-white solid. Mp 175.8–177.6 °C (165−168 °C [32]). ^1^H NMR (600 MHz, DMSO-*d*_6_) *δ* 9.57 (s, 1H), 8.18 (d, *J* = 7.8 Hz, 2H), 8.09 (dd, *J* = 7.8, 1.1 Hz, 1H), 7.78 (td, *J* = 8.4, 1.3 Hz, 1H), 7.73 (d, *J* = 8.4 Hz, 1H), 7.54 (t, *J* = 7.3 Hz, 1H), 7.54 (t, *J* = 7.3 Hz, 2H), 7.48 (t, *J* = 7.3 Hz, 1H), 7.54 (t, *J* = 7.3 Hz, 1H). ^13^C NMR (150 MHz, DMSO-d6) *δ* 173.3, 154.8, 145.5, 139.2, 134.0, 131.4, 130.2, 128.8, 127.9, 125.0, 124.9, 121.5, 118.6. LCMass for C_15_H_9_O_3_ [M − H]^+^ 237.23. Found: 237.33. Purity: 96.7%.

#### 3.3.4. 2-(2-Fluorophenyl)-3-hydroxy-4H-chromen-4-one (**6b**)

Yield: 77% (49% [32]). A pink solid. Mp 176.0−181.3 °C (181−182 °C [32]). ^1^H NMR (600 MHz, DMSO-*d*_6_) *δ* 9.40 (s, 1H), 8.13 (d, *J* = 8.0 Hz, 1H), 7.77 (td, *J* = 8.5, 1.3 Hz, 1H), 7.74 (t, *J* = 7.4 Hz, 1H), 7.62 (d, *J* = 8.5 Hz, 1H), 7.59 (ddd, *J* = 12.8, 7.0, 1.3 Hz, 1H), 7.47 (t, *J* = 7.4 Hz, 1H), 7.37 (dd, *J* = 6.9, 4.4 Hz, 1H), 7.36 (d, *J* = 8.0 Hz, 1H). ^13^C NMR (150 MHz, DMSO-*d*_6_) *δ* 173.0, 159.4 (^1^*J* = 250.5 Hz), 155.2, 143.7, 139.7, 134.1, 132.8 (^3^*J* = 9.0 Hz), 131.4, 125.2, 125.0 (^2^*J* = 25.5 Hz), 124.7, 122.0, 119.2, 119.1, 118.6, 116.4 (^2^*J* = 21.0 Hz). LCMass for C_15_H_10_FO_3_ [M + H]^+^ 257.24. Found: 257.17. Purity: 99.9%.

#### 3.3.5. 2-(2-Chlorophenyl)-3-hydroxy-4H-chromen-4-one (**6c**)

Yield: 70% (67% [32]). A yellow-white solid. Mp 187.1−188.1 °C (177−179 °C [32]). ^1^H NMR (600 MHz, DMSO-*d*_6_) *δ* 9.34 (s, 1H), 8.14 (dd, *J* = 8.0, 1.6 Hz, 1H), 7.78 (dd, *J* = 8.3, 1.6 Hz, 1H), 7.67 (dd, *J* = 7.6, 1.6 Hz, 1H), 7.62 (t, *J* = 8.3 Hz, 2H), 7.55 (td, *J* = 8.0, 1.6 Hz, 1H), 7.49 (t, *J* = 7.7 Hz, 1H), 7.48 (t, *J* = 7.7 Hz, 1H). ^13^C NMR (150 MHz, DMSO-*d*_6_) d 173.0, 155.0, 146.2, 139.2, 133.9, 132.7, 132.0, 131.9, 130.0, 129.8, 127.3, 125.0, 124.8, 121.9, 118.4. LCMass for C_15_H_10_^35^ClO_3_ [M + H]^+^ 273.21. Found: 273.17. Purity: 98.2%.

#### 3.3.6. 2-(2-Bromophenyl)-3-hydroxy-4*H*-chromen-4-one (**6d**)

Yield: 76%. A yellow-white solid. Mp 184.4−186.3 °C. ^1^H NMR (600 MHz, DMSO-*d*_6_) *δ* 9.31 (s, 1H), 8.15 (d, *J* = 7.8 Hz, 1H), 7.81−7.77 (m, 2H), 7.65 (d, *J* = 7.8 Hz, 1H), 7.63 (d, *J* = 8.4 Hz, 1H), 7.54 (t, *J* = 7.8 Hz, 1H), 7.48 (t, *J* = 8.4 Hz, 1H), 7.47 (dd, *J* = 7.8, 1.2 Hz, 1H). ^13^C NMR (150 MHz, DMSO-*d*_6_) *δ* 173.1, 154.9, 147.5, 139.0, 133.9, 132.9, 132.1, 132.0, 127.8, 124.9 (x2), 124.8, 122.6, 122.0, 118.4. LCMass for C_15_H_10_^79^BrO_3_ [M + H]^+^ 317.16. Found: 317.08. Purity: 99.9%.

#### 3.3.7. 3-Hydroxy-2-(3-methoxyphenyl)-4*H*-chromen-4-one (**6e**)

Yield: 72% (39.2% [33]). A white solid. Mp 132.5−134.0 °C (133.0–135.0 °C [33]). ^1^H NMR (600 MHz, DMSO-*d*_6_) *δ* 9.59 (s, 1H), 8.09 (dd, *J* = 7.9, 1.1 Hz, 1H), 7.78 (td, *J* = 7.6, 1.6 Hz, 2H), 7.74 (t, *J* = 7.9 Hz, 1H), 7.44 (d, *J* = 7.0 Hz, 1H), 7.07 (dd, *J* = 8.2, 2.5 Hz, 1H), 3.81 (s, 3H). ^13^C NMR (150 MHz, DMSO-*d*_6_) *δ* 173.3, 159.4, 154.8, 145.2, 139.4, 134.0, 132.7, 129.9, 125.0, 124.9, 121.4, 120.3, 118.7, 115.5, 113.6, 55.5. LCMass for C_16_H_13_O_4_ [M + H]^+^ 269.27. Found: 269.25. Purity: 97.8%.

#### 3.3.8. 2-(3-Fluorophenyl)-3-hydroxy-4*H*-chromen-4-one (**6f**)

Yield: 62% (50% [32]). A yellow-white solid. Mp 172.3−176.4 °C (171−173 °C [32]). ^1^H NMR (600 MHz, DMSO-*d*_6_) *δ* 9.86 (s, 1H), 8.09 (d, *J* = 7.9 Hz, 1H), 8.06 (d, *J* = 7.9 Hz, 1H), 7.99 (d, *J* = 11.0 Hz, 1H), 7.79 (td, *J* = 8.0, 1.0 Hz, 1H), 7.76 (d, *J* = 8.3 Hz, 1H), 7.60 (dd, *J* = 14.4, 7.8 Hz, 1H), 7.46 (t, *J* = 7.8 Hz, 1H), 7.32 (td, *J* = 9.0, 3.0 Hz, 1H). ^13^C NMR (150 MHz, DMSO-*d*_6_) *δ* 173.2, 162.0 (^1^*J* = 240.0 Hz), 154.5, 143.6, 139.5, 134.0, 133.4 (^3^*J* = 7.5 Hz), 130.7 (^3^*J* = 9.0 Hz), 124.8 (x2), 123.7, 121.2, 118.5, 116.7 (^2^*J* = 21.0), 114.2 (^2^*J* = 24.0 Hz). LCMass for C_15_H_10_FO_3_ [M + H]^+^ 257.24. Found: 257.17. Purity: 97.5%.

#### 3.3.9. 2-(3-Chlorophenyl)-3-hydroxy-4*H*-chromen-4-one (**6g**)

Yield: 69% (29% [32]). A yellow-white solid. Mp 156.8−160.9 °C (157−159 °C [32]). ^1^H NMR (600 MHz, DMSO-*d*_6_) *δ* 9.88 (s, 1H), 8.21 (s, 1H), 8.13 (d, *J* = 7.8 Hz, 1H), 8.07 (d, *J* = 8.0 Hz, 1H), 7.77 (t, *J* = 8.0 Hz, 1H), 7.73 (d, *J* = 8.4 Hz, 1H), 7.56 (t, *J* = 7.9 Hz, 1H), 7.52 (d, *J* = 7.3 Hz, 1H), 7.44 (t, *J* = 7.3 Hz, 1H). ^13^C NMR (150 MHz, DMSO-*d*_6_) *δ* 173.4, 154.8, 143.7, 139.8, 134.3, 133.6, 133.5, 130.7, 129.8, 127.3, 126.3, 125.0, 124.9, 121.4, 118.7. LCMass for C_15_H_10_^35^ClO_3_ [M + H]^+^ 273.21. Found: 273.08. Purity: 99.9%.

#### 3.3.10. 2-(3-Bromophenyl)-3-hydroxy-4*H*-chromen-4-one (**6h**)

Yield: 60% (53.5% [33]). A yellow-white solid. Mp 167.3−172.0 °C (162−164 °C [33]). ^1^H NMR (600 MHz, DMSO-d6) *δ* 9.90 (s, 1H), 8.39 (s, 1H), 8.21 (d, *J* = 8.4 Hz, 1H), 8.11 (d, *J* = 7.8 Hz, 1H), 7.80 (t, *J* = 6.6 Hz, 1H), 7.79 (d, *J* = 8.4 Hz, 1H), 7.69 (d, *J* = 7.8 Hz, 1H), 7.53 (t, *J* = 7.8 Hz, 1H), 7.47 (t, *J* = 6.6 Hz, 1H). ^13^C NMR (150 MHz, DMSO-*d*_6_) *δ* 173.1, 154.6, 143.3, 139.7, 134.0, 133.6, 132.5, 130.8, 129.9, 126.4, 124.8, 124.7, 121.9, 121.3, 118.6. LCMass for C_15_H_10_^79^BrO_3_ [M + H]^+^ 317.16. Found: 317.08. Purity: 99.9%.

#### 3.3.11. 3-Hydroxy-2-(4-methoxyphenyl)-4*H*-chromen-4-one (**6i**)

Yield: 78% (74.6% [33]). A yellow-white solid. Mp 240.9−244.8 °C (234−236 °C [33]). ^1^H NMR (600 MHz, DMSO-*d*_6_) *δ* 9.41 (s, 1H), 8.19 (d, *J* = 9.0 Hz, 2H), 8.09 (d, *J* = 7.8 Hz, 1H), 7.77 (td, *J* = 7.2, 1.2 Hz, 1H), 7.73 (d, *J* = 8.4 Hz, 1H), 7.45 (t, *J* = 7.2 Hz, 1H), 7.12 (d, *J* = 9.0 Hz, 2H), 3.83 (s, 3H). ^13^C NMR (150 MHz, DMSO-*d*_6_) *δ* 172.7, 160.5, 154.4, 145.7, 138.1, 133.6, 129.5 (x2), 124.7, 124.6, 123.5, 121.3, 118.3, 114.1 (x2), 55.4. LCMass for C_16_H_13_O_4_ [M + H]^+^ 269.27. Found: 269.17. Purity: 95.5%.

#### 3.3.12. 2-(4-Fluorophenyl)-3-hydroxy-4*H*-chromen-4-one (**6j**)

Yield: 59% (44% [32]). A white solid. Mp 155.5−155.8 °C (151−152 °C [32]). ^1^H NMR (600 MHz, DMSO-*d*_6_) *δ* 9.64 (s, 1H), 8.24 (dd, *J* = 8.5, 8.5 Hz, 2H), 8.08 (d, *J* = 7.9 Hz, 1H), 7.78 (t, *J* = 7.4 Hz, 1H), 7.72 (d, *J* = 8.5 Hz, 1H), 7.45 (t, *J* = 7.4 Hz, 1H), 7.37 (t, *J* = 8.5 Hz, 2H). ^13^C NMR (150 MHz, DMSO-*d*_6_) *δ* 173.2, 162.8 (^1^*J* = 247.5 Hz), 154.7, 144.7, 139.0, 134.0, 130.3 (^3^*J* = 9.0 Hz), 127.9, 125.0, 124.9, 121.4, 118.6, 115.8 (^2^*J* = 22.5 Hz). LCMass for C_15_H_10_FO_3_ [M + H]^+^ 257.24. Found: 257.17. Purity: 92.2%.

#### 3.3.13. 2-(4-Chlorophenyl)-3-hydroxy-4*H*-chromen-4-one (**6k**)

Yield: 83% (58% [32]). A white solid. Mp 155.5−155.8 °C (202−204 °C [32]). ^1^H NMR (600 MHz, DMSO-*d*_6_) *δ* 9.78 (s, 1H), 8.21 (d, *J* = 8.6 Hz, 2H), 8.08 (dd, *J* = 7.4, 1.1 Hz, 1H), 7.78 (td, *J* = 8.4, 1.4 Hz, 1H), 7.72 (d, *J* = 8.4 Hz, 1H), 7.60 (d, *J* = 8.6 Hz, 2H), 7.45 (t, *J* = 7.4 Hz, 1H). ^13^C NMR (150 MHz, DMSO-*d*_6_) *δ* 173.3, 154.7, 144.3, 139.5, 134.7, 134.1, 130.3, 129.6, 128.9, 125.0, 124.9, 121.4, 118.6. LCMass for C_15_H_10_^35^ClO_3_ [M + H]^+^ 273.21. Found: 273.08. Purity: 99.2%.

#### 3.3.14. 2-(4-Bromophenyl)-3-hydroxy-4*H*-chromen-4-one (**6l**)

Yield: 78 % (58% [32]). A yellow-white solid. Mp 205.0−209.5 °C (163−167 °C [32]). ^1^H NMR (600 MHz, DMSO-*d*_6_) *δ* 9.78 (s, 1H), 8.14 (d, *J* = 8.6 Hz, 2H), 8.09 (dd, *J* = 8.0, 1.4 Hz, 1H), 7.79 (td, *J* = 8.5, 1.4 Hz, 1H), 7.74 (d, *J* = 8.6 Hz, 2H), 7.73 (d, *J* = 8.0 Hz, 1H), 7.46 (t, *J* = 7.3 Hz, 1H). ^13^C NMR (150 MHz, DMSO-*d*_6_) *δ* 173.3, 154.7, 144.3, 139.5, 134.2, 131.8, 130.7, 129.7, 125.0, 124.9, 123.6, 121.4, 118.6. LCMass for C_15_H_10_^79^BrO_3_ [M + H]^+^ 317.16. Found: 317.00. Purity: 99.9%.

#### 3.3.15. 6-Bromo-3-hydroxy-2-(4-methoxyphenyl)-4*H*-chromen-4-one (**7i**)

Yield: 84%. A yellow solid. Mp 197.9−204.9 °C. ^1^H NMR (600 MHz, DMSO-*d*_6_) *δ* 9.60 (s, 1H), 8.16 (d, *J* = 8.9 Hz, 2H), 8.12 (d, *J* = 2.5 Hz, 1H), 7.88 (dd, *J* = 9.0, 2.5 Hz, 1H), 7.70 (d, *J* = 9.0 Hz, 1H), 7.09 (d, *J* = 8.9 Hz, 2H), 3.82 (s, 3H). ^13^C NMR (150 MHz, DMSO-*d*_6_) *δ* 171.7, 160.9, 153.5, 146.6, 138.5, 136.3, 129.8, 126.9, 123.4, 123.1, 117.1, 114.3, 55.6. LCMass for C_16_H_11_^79^BrO_4_ [M + H]^+^ 347.19. Found: 347.17. Purity: 97.6%.

#### 3.3.16. 6-Bromo-2-(4-fluorophenyl)-3-hydroxy-4*H*-chromen-4-one (**7j**)

Yield: 77% (70% [34]). A yellow solid. Mp 212.5−215.9 °C (188-190 °C [34]). ^1^H NMR (600 MHz, DMSO-*d*_6_) *δ* 9.81 (s, 1H), 8.20 (dd, *J*= 8.9, 5.6 Hz, 2H), 8.09 (d, *J* = 2.4 Hz, 1H), 7.86 (dd, *J* = 8.9, 2.4 Hz, 1H), 7.67 (d, *J* = 8.9 Hz, 1H), 7.34 (t, *J* = 8.9 Hz, 2H). ^13^C NMR (150 MHz, DMSO-*d*_6_) *δ* 172.0, 162.9 (^1^*J* = 248.1 Hz), 153.5, 145.2, 139.1, 136.4, 130.4 (^3^*J* = 8.4 Hz), 127.6, 126.9, 123.0, 121.3, 117.1, 115.8 (^2^*J* = 21.6 Hz). LCMass for C_15_H_7_BrFO_3_ [M − H]^+^ 333.13. Found: 333.17. Purity: 99.9%.

#### 3.3.17. 6-Bromo-2-(4-chlorophenyl)-3-hydroxy-4*H*-chromen-4-one (**7k**)

Yield: 89% (85% [34]). A yellow solid. Mp 217.5−220.5 °C (172−173 °C [34]). ^1^H NMR (600 MHz, DMSO-*d*_6_) *δ* 9.98 (s, 1H), 8.20 (d, *J* = 8.8 Hz, 2H), 8.13 (d, *J* = 2.5 Hz, 1H), 7.91 (dd, *J* = 8.8, 2.5 Hz, 1H), 7.72 (d, *J* = 8.8 Hz, 1H), 7.60 (d, *J* = 8.8 Hz, 2H). ^13^C NMR (150 MHz, DMSO-*d*_6_) *δ* 171.9, 153.4, 144.7, 139.4, 136.4, 134.7, 129.8, 129.4, 128.7, 126.7, 122.8, 121.2, 117.0. LCMass for C_15_H_9_BrClO_3_ [M + H]^+^ 351.12. Found: 351.08. Purity: 99.9%.

#### 3.3.18. 6-Bromo-2-(4-bromophenyl)-3-hydroxy-4*H*-chromen-4-one (**7l**)

Yield: 91% (77% [34]). A yellow-white solid. Mp 248.5−249.6 °C (233−235 °C [34]). ^1^H NMR (600 MHz, DMSO-*d*_6_) *δ* 10.02 (s, 1H), 8.15 (s, 1H), 8.14 (d, *J* = 8.7 Hz, 2H), 7.93 (dd, *J* = 9.0, 2.5 Hz, 1H), 7.75 (d, *J* = 8.7 Hz, 2H), 7.74 (d, *J* = 9.0 Hz, 1H). ^13^C NMR (150 MHz, DMSO-*d*_6_) *δ* 172.0, 153.5, 144.8, 139.6, 136.5, 131.7, 130.3, 129.7, 126.9, 123.7, 123.0, 121.3, 117.1. LCMass for C_15_H_8_Br_2_O_3_ [M + H]^+^ 395.07. Found: 395.00. Purity: 99.9%.

### 3.4. Pharmacological/Biological Assays

#### 3.4.1. Cell Culture

Prof. Y. Su kindly provided human non-small lung cancer cells (A549 cells) from the National Yang Ming Chiao Tung University, Taipei, Taiwan. The cells were stored in liquid nitrogen (−196 °C). After the cells were thawed, they were incubated at 37 °C in CO_2_ (5%) and cultured in Dulbecco’s Modified Eagle Medium (DMEM) containing fetal bovine serum (10%, FBS), penicillin (100 U/mL), streptomycin (100 μg/mL), L-glutamine (2 μM), and sodium pyruvate (1 mM). The cells were passaged twice weekly, and the experiment was completed within 30 generations to minimize experimental errors [31,35].

#### 3.4.2. In Vitro Cytotoxicity Assay

Cell viability was evaluated using the MTT assay [35,36] to further assess cytotoxicity. The compound stock solution was stored in dimethyl sulfoxide (DMSO) at a concentration of 100 mM at −20 °C and thawed immediately before use. Briefly, the cells were incubated in 96-well culture plates (3 × 10^3^ cells in 200 μL per well). After 24 h, cells were treated with different concentrations (3.125, 6.25, 12.5, 25, 50, and 100 μM) of all 16 synthesized compounds, and 5-FU was used as the positive control. After 72 h, the attached cells were treated with an MTT reagent (0.5 mg/mL to 100 μL of each well) and incubated at 37 °C for 3 h. This reagent was then removed, DMSO (100 μL) was added to each well to dissolve the formazan metabolite, and the amount of formazan was quantified by measuring the absorbance at 570 nm using an ELISA plate reader (TECAN Spark, Tecan Group Ltd., Männedorf, Switzerland) (μ Quant). The optical density of formazan formed in the control (untreated) cells was 100% viability.

#### 3.4.3. Western Blotting Analysis

Western blotting was performed according to a previously described method [35,36]. Briefly, the cells were seeded in 6-well culture plates. After reaching 85–90% confluence, cells were treated with **6l** (0.25, 0.5, 1, and 2 μM) and 5-FU (5 μM) followed by incubation for 48 h. The cells were then collected and lysed using a radioimmunoprecipitation assay (RIPA) buffer. Lysates of the total protein were separated on sodium dodecyl sulfate polyacrylamide gels (12.5%) and transferred to polyvinylidene difluoride membranes. The membranes were blocked with a bovine serum albumin (2%, BSA) solution and incubated with anti-Bax, anti-Bcl-2 (Cell Signaling Inc., Danvers, MA, USA), anti-caspase-3, and anti-β-actin (GeneTex Inc., Irvine, CA, USA) primary antibodies at 4 °C overnight. Each membrane was washed three times with Tris-buffered saline containing Tween 20 (0.1%, TBST) and incubated with horseradish peroxidase (HRP)-conjugated secondary antibodies at room temperature for 2 h. Finally, the membranes were developed using an enhanced chemiluminescence (ECL) detection kit and visualized using an ImageQuant LAS 4000 Mini bio-molecular imager (GE Healthcare, Marlborough, MA, USA). Band densities were quantified using ImageJ software 1.53a (BioTechniques, New York, NY, USA).

#### 3.4.4. Statistical Analysis

All results are presented as mean ± SEM. Statistical analysis was executed using Student’s *t*-test. A probability of 0.05 or less was considered to be statistically significant. Microsoft Excel 2019 was used for the statistical and graphical assessment. All experiments were executed at least 3 times.

## 4. Conclusions

**Figure 4:**
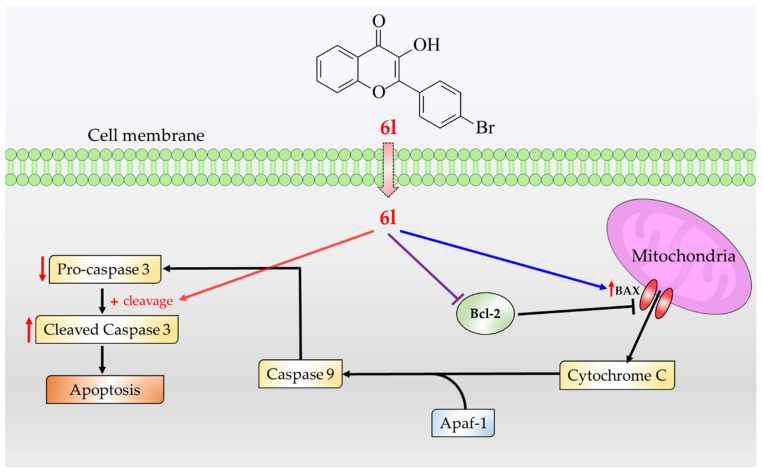
Schematic diagram for cancer cell apoptosis mechanism of compound **6l** in A549 cells.

In conclusion, among the sixteen synthesized flavonoids, 2-(4-bromophenyl)-3-hydroxy-4*H*-chromen-4-one (**6l**) exhibited the highest cytotoxic activity against A549 cells. Furthermore, compounds **6a**, **6j**, and **6k** exhibited activities comparable to 5-FU. The structure–activity relationship studies revealed that the halogenation at C’-4 in the B ring enhanced the cytotoxic effects against A549 cells. The Western blot analysis confirmed that compound **6l** induced apoptosis in A549 cells via mitochondrial- and caspase-3-dependant pathways (Figure 4). The present study suggests that bioactive synthetic compounds **6a**, **6j**, **6k**, and **6l** (especially **6l**), are potential cytotoxic agents and could be promising candidates for developing novel anticancer drugs.

## Figures and Tables

**Table 1:** Inhibitory effects of compounds against human non-small cell lung cancer cells (A549).

Compounds	IC_50_ (μM) ^a^
**6a**	6.34 ± 0.89 **
**6b**	38.17 ± 4.21 *
**6c**	24.54 ± 3.13 *
**6d**	22.97 ± 2.73 *
**6e**	15.07 ± 0.93 **
**6f**	14.64 ± 1.48 *
**6g**	10.78 ± 0.97 **
**6h**	8.25 ± 0.77 **
**6i**	>100
**6j**	6.13 ± 0.63 **
**6k**	3.14 ± 0.29 **
**6l**	0.46 ± 0.02 ***
**7i**	22.04 ± 3.29 *
**7j**	19.44 ± 1.82 *
**7k**	31.35 ± 6.64 *
**7l**	47.58 ± 7.11 *
5-FU ^b^	4.98 ± 0.41 **

Results are presented as averages ± SD (n = 3). ^a^ Concentration necessary for 50% inhibition (IC_50_). ^b^ 5-Flurouracie (5-FU) was used as a positive control; *** *p* < 0.001, ** *p* < 0.01, and * *p* < 0.05 compared with the control.

## Data Availability

The data presented in this study are available in the main text and the Supplementary Materials of this article.

## Supplementary Materials

The following are available online at https://www.mdpi.com/article/10.3390/molecules29092041/s1. Figure S1: ^1^H NMR (600 MHz, DMSO-*d*_6_) for compound **6a**. Figure S2: ^13^C NMR (150 MHz, DMSO-*d*_6_) for compound **6a**. Figure S3: ^1^H NMR (600 MHz, DMSO-*d*_6_) for compound **6b**. Figure S4: ^13^C NMR (150 MHz, DMSO-*d*_6_) for compound **6b**. Figure S5: ^1^H NMR (600 MHz, DMSO-*d*_6_) for compound **6c**. Figure S6: ^13^C NMR (150 MHz, DMSO-*d*_6_) for compound **6c**. Figure S7: ^1^H NMR (600 MHz, DMSO-*d*_6_) for compound **6d**. Figure S8: ^13^C NMR (150 MHz, DMSO-*d*_6_) for compound **6d**. Figure S9: ^1^H NMR (600 MHz, DMSO-*d*_6_) for compound **6e**. Figure S10: ^13^C NMR (150 MHz, DMSO-*d*_6_) for compound **6e**. Figure S11: ^1^H NMR (600 MHz, DMSO-*d*_6_) for compound **6f**. Figure S12: ^13^C NMR (150 MHz, DMSO-*d*_6_) for compound **6f**. Figure S13: ^1^H NMR (600 MHz, DMSO-*d*_6_) for compound **6g**. Figure S14: ^13^C NMR (150 MHz, DMSO-*d*_6_) for compound **6g**. Figure S15: ^1^H NMR (600 MHz, DMSO-*d*_6_) for compound **6h**. Figure S16: ^13^C NMR (150 MHz, DMSO-*d*_6_) for compound **6h**. Figure S17: ^1^H NMR (600 MHz, DMSO-*d*_6_) for compound **6i**. Figure S18: ^13^C NMR (150 MHz, DMSO-*d*_6_) for compound **6i**. Figure S19: ^1^H NMR (600 MHz, DMSO-*d*_6_) for compound **6j**. Figure S20: ^13^C NMR (150 MHz, DMSO-*d*_6_) for compound **6j**. Figure S21: ^1^H NMR (600 MHz, DMSO-*d*_6_) for compound **6k**. Figure S22: ^13^C NMR (150 MHz, DMSO-*d*_6_) for compound **6k**. Figure S23: ^1^H NMR (600 MHz, DMSO-*d*_6_) for compound **6l**. Figure S24: ^13^C NMR (150 MHz, DMSO-*d*_6_) for compound **6l**. Figure S25: ^1^H NMR (600 MHz, DMSO-*d*_6_) for compound **7i**. Figure S26: ^13^C NMR (150 MHz, DMSO-*d*_6_) for compound **7i**. Figure S27: ^1^H NMR (600 MHz, DMSO-*d*_6_) for compound **7j**. Figure S28: ^13^C NMR (150 MHz, DMSO-*d*_6_) for compound **7j**. Figure S29: ^1^H NMR (600 MHz, DMSO-*d*_6_) for compound **7k**. Figure S30: ^13^C NMR (150 MHz, DMSO-*d*_6_) for compound **7k**. Figure S31: ^1^H NMR (600 MHz, DMSO-*d*_6_) for compound **7l**. Figure S32: ^13^C NMR (150 MHz, DMSO-*d*_6_) for compound **7l**.
