# Supplementary material for: Synthesis of Flavonols and Assessment of Their Biological Activity as Anticancer Agents
Source: Molecules. 2024 Apr 28;29(9):2041. doi: 10.3390/molecules29092041 (PMC11085485; doi:10.3390/molecules29092041)
Supplement: Supplementary file 1 [file molecules-29-02041-s001.zip › molecules-2930553-supplementary.pdf]

Supplementary data

Synthesis of Flavonols and Assessment of Their Biological Activities as Anticancer Agents

Yu-Hui Hsieh [1], Pei-Hsuan Hsu [2], Anren Hu [3], Yang-Je Cheng [2], Tzenge-Lien Shih [2,*] and Jih-Jung Chen [1,4,5,6,*]

[1] Biomedical Industry Ph.D. Program, School of Life Sciences, National Yang Ming Chiao Tung University, Taipei 112304, Taiwan; hsieh.ls10@nycu.edu.tw (Y.-H.H.)
[2] Department of Chemistry, Tamkang University, New Taipei City 251301, Taiwan; peggy060708@gmail.com (P.-H.H.); jason0110422@gmail.com (Y.-J.C.)
[3] Department of Laboratory Medicine and Biotechnology, Tzu Chi University, Hualien 970374, Taiwan; anren@mail.tcu.edu.tw (A.H.)
[4] Department of Pharmacy, School of Pharmaceutical Sciences, National Yang Ming Chiao Tung University, Taipei 112304, Taiwan
[5] Department of Medical Research, China Medical University Hospital, China Medical University, Taichung 404333, Taiwan
[6] Traditional Herbal Medicine Research Center, Taipei Medical University Hospital, Taipei 110301, Taiwan
* Correspondence: jjungchen@nycu.edu.tw (J.-J.C.)*; Tel.: +886-2-2826-7195 (J.-J.C.)*; Fax: +886-2-2823-2940(J.-J.C.)*; tlshih@mail.tku.edu.tw (T.-L.S.)*;Tel.: +886-2-8631-5024 (T.-L.S.)*

Contents

Figure S1. ^{1}H NMR (600 MHz, DMSO-d_6) for compound **6a**……………………………………S4

Figure S2. ^{13}C NMR (150 MHz, DMSO-d_6) for compound **6a**……………………………………S5

Figure S3. ^{1}H NMR (600 MHz, DMSO-d_6) for compound **6b**……………………………………S6

Figure S4. ^{13}C NMR (150 MHz, DMSO-d_6) for compound **6b**……………………………………S7

Figure S5. ^{1}H NMR (600 MHz, DMSO-d_6) for compound **6c**……………………………………S8

Figure S6. ^{13}C NMR (150 MHz, DMSO-d_6) for compound **6c**……………………………………S9

Figure S7. ^{1}H NMR (600 MHz, DMSO-d_6) for compound **6d**……………………………………S10

Figure S8. ^{13}C NMR (150 MHz, DMSO-d_6) for compound **6d**……………………………………S11

Figure S9. ^{1}H NMR (600 MHz, DMSO-d_6) for compound **6e**……………………………………S12

Figure S10. ^{13}C NMR (150 MHz, DMSO-d_6) for compound **6e**……………………………………S13

Figure S11. ^{1}H NMR (600 MHz, DMSO-d_6) for compound **6f**……………………………………S14

Figure S12. ^{13}C NMR (150 MHz, DMSO-d_6) for compound **6f**……………………………………S15

Figure S13. ^{1}H NMR (600 MHz, DMSO-d_6) for compound **6g**……………………………………S16

Figure S14. ^{13}C NMR (150 MHz, DMSO-d_6) for compound **6g**……………………………………S17

Figure S15. ^{1}H NMR (600 MHz, DMSO-d_6) for compound **6h**……………………………………S18

Figure S16. ^{13}C NMR (150 MHz, DMSO-d_6) for compound **6h**……………………………………S19

Figure S17. ^{1}H NMR (600 MHz, DMSO-d_6) for compound **6i**……………………………………S20

Figure S18. ^{13}C NMR (150 MHz, DMSO-d_6) for compound **6i**……………………………………S21

Figure S19. ^{1}H NMR (600 MHz, DMSO-d_6) for compound **6j**……………………………………S22

Figure S20. ^{13}C NMR (150 MHz, DMSO-d_6) for compound **6j**………………………………..……..**S23**

Figure S21. ^{1}H NMR (600 MHz, DMSO-d_6) for compound **6k**……………..………………………**S24**

Figure S22. ^{13}C NMR (150 MHz, DMSO-d_6) for compound **6k**…………………………….………**S25**

Figure S23. ^{1}H NMR (600 MHz, DMSO-d_6) for compound **6l**…………………………………………**S26**

Figure S24. ^{13}C NMR (150 MHz, DMSO-d_6) for compound **6l**………………………………..……..**S27**

Figure S25. ^{1}H NMR (600 MHz, DMSO-d_6) for compound **7i**…………………………………………**S28**

Figure S26. ^{13}C NMR (150 MHz, DMSO-d_6) for compound **7i**………………………………..……..**S29**

Figure S27. ^{1}H NMR (600 MHz, DMSO-d_6) for compound **7j**…………………………………………**S30**

Figure S28. ^{13}C NMR (150 MHz, DMSO-d_6) for compound **7j**…………………………..…………**S31**

Figure S29. ^{1}H NMR (600 MHz, DMSO-d_6) for compound **7k**……………..………………………**S32**

Figure S30. ^{13}C NMR (150 MHz, DMSO-d_6) for compound **7k**…………………………….………**S33**

Figure S31. ^{1}H NMR (600 MHz, DMSO-d_6) for compound **7l**…………………………………………**S34**

Figure S32. ^{13}C NMR (150 MHz, DMSO-d_6) for compound **7l**…………………………,……….**S35**

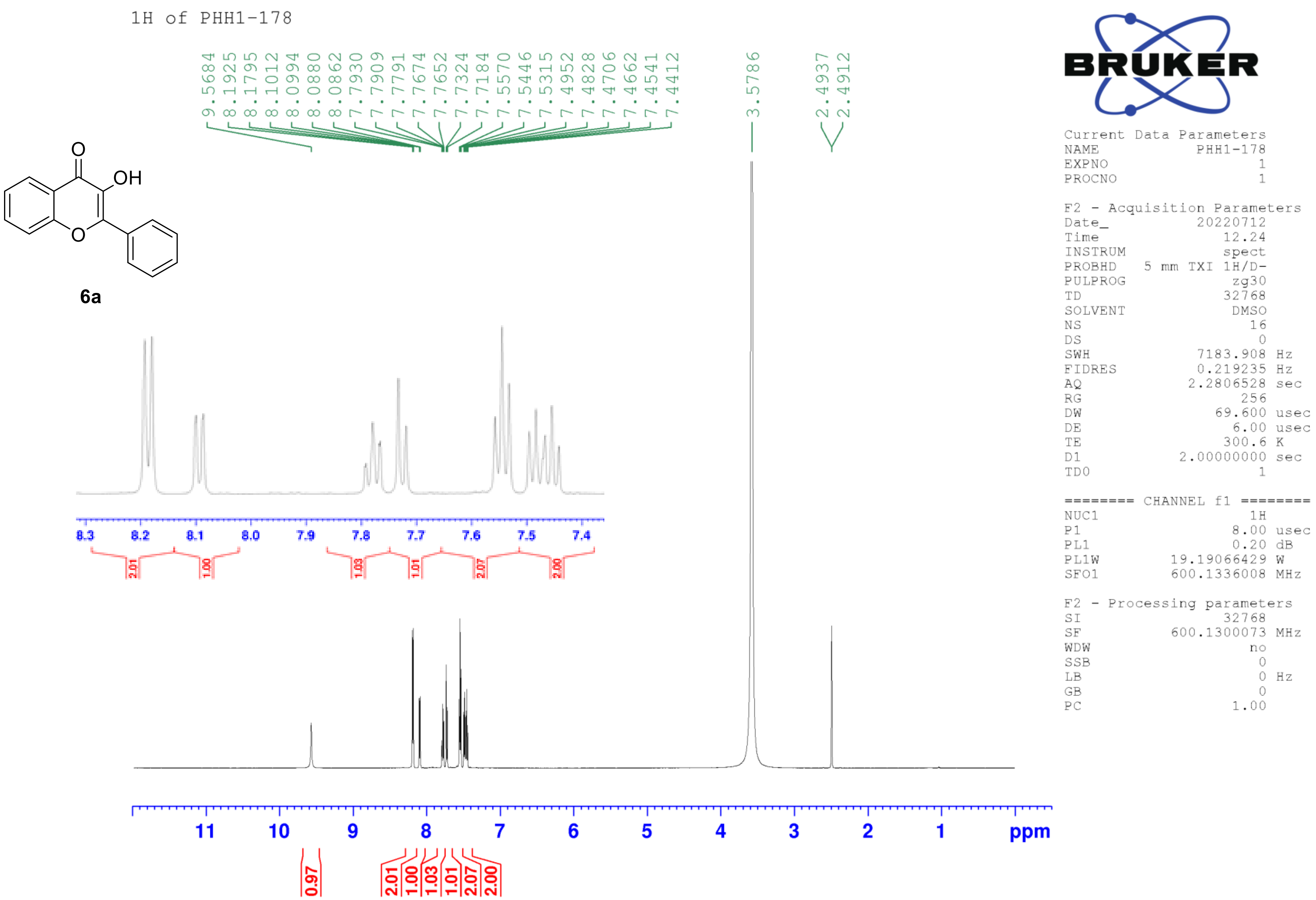

Figure S1. ^{1}H NMR (600 MHz, DMSO-d_6) for compound **6a**.

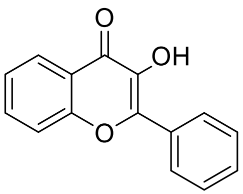

6a

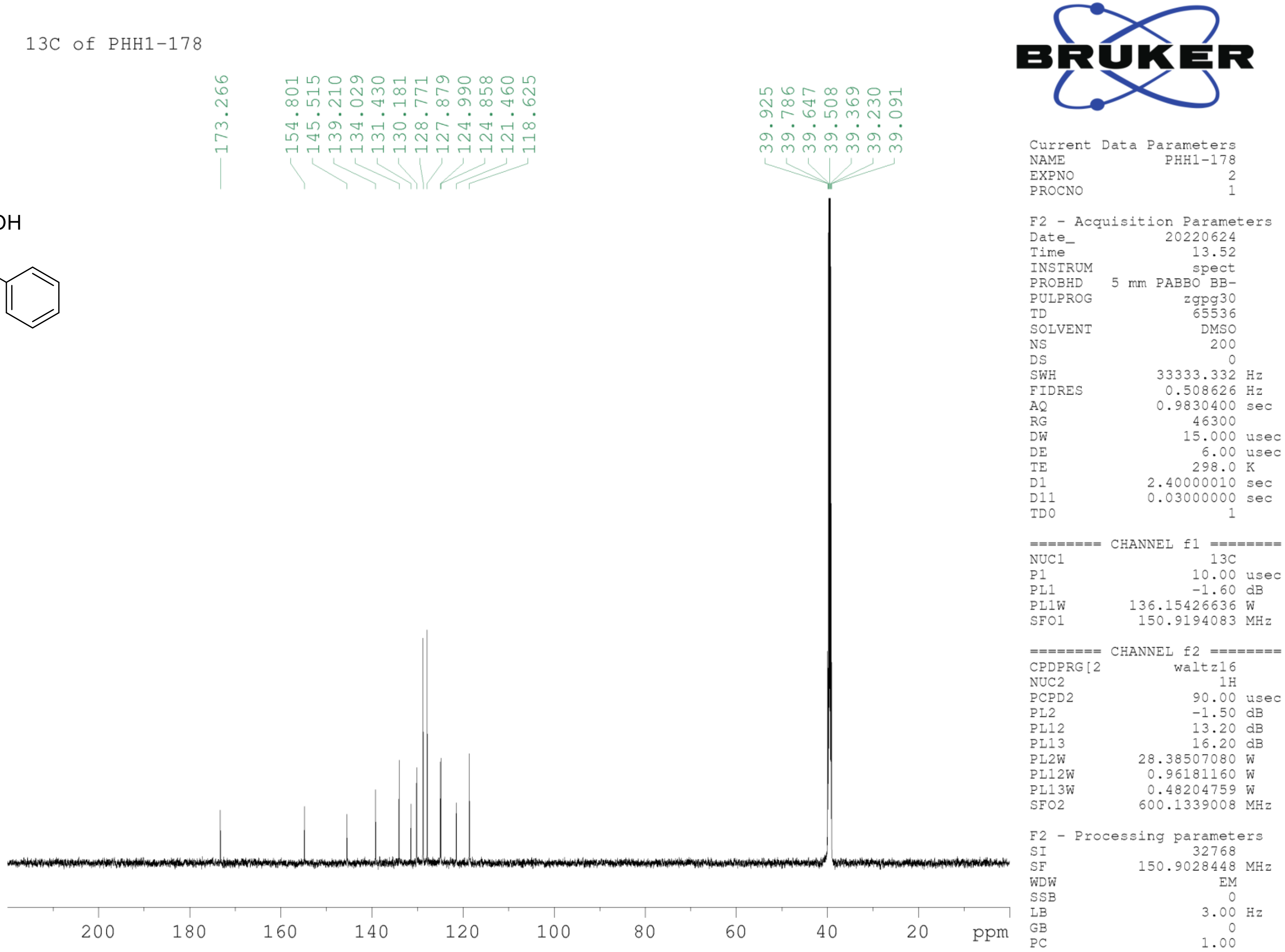

Figure S2. ^{13}C NMR (150 MHz, DMSO-d_6) for compound **6a**.

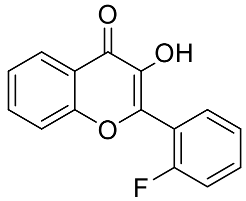

6b

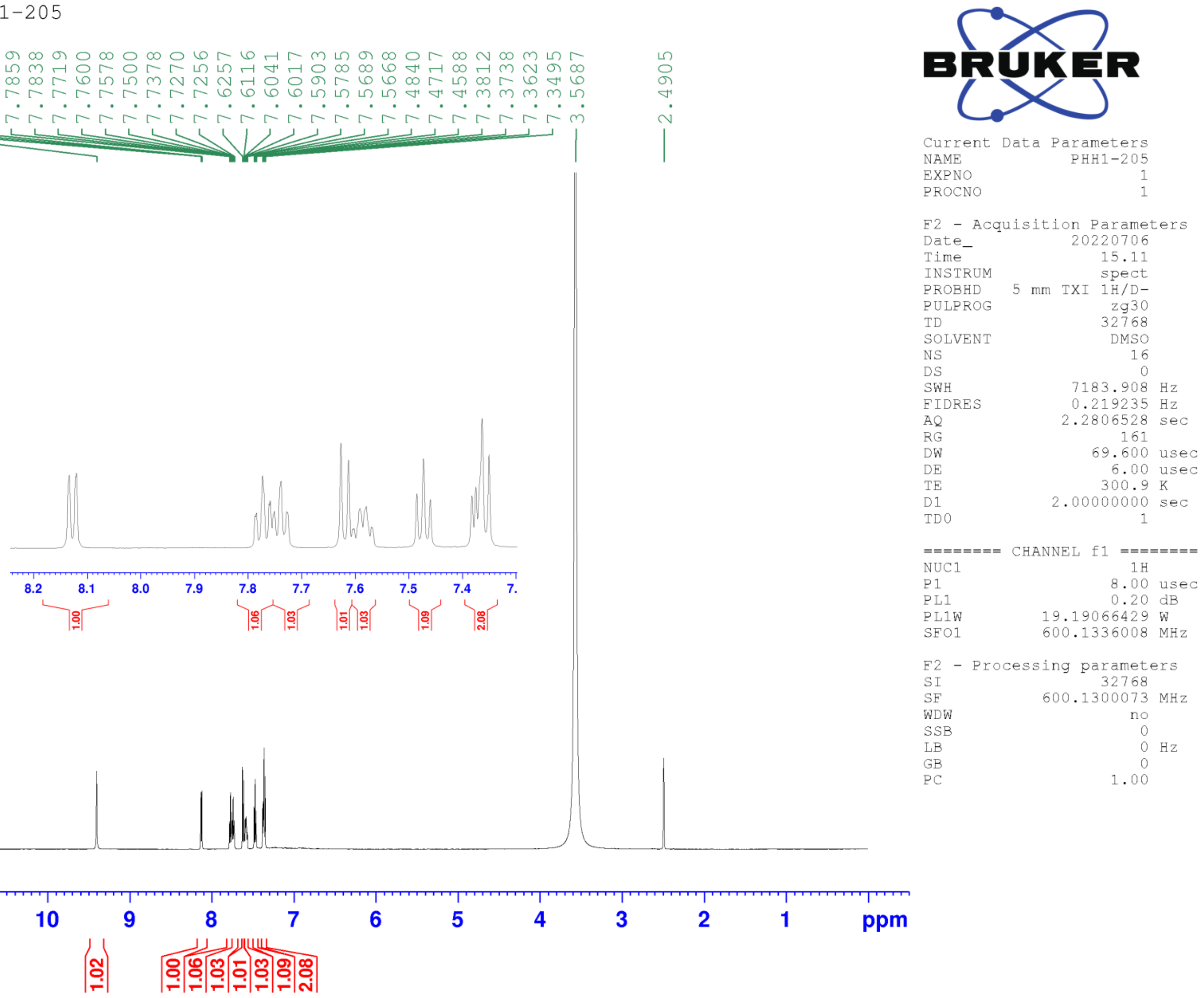

Figure S3. ^{1}H NMR (600 MHz, DMSO-d_6) for compound **6b**.

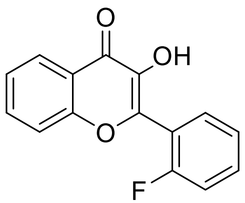

6b

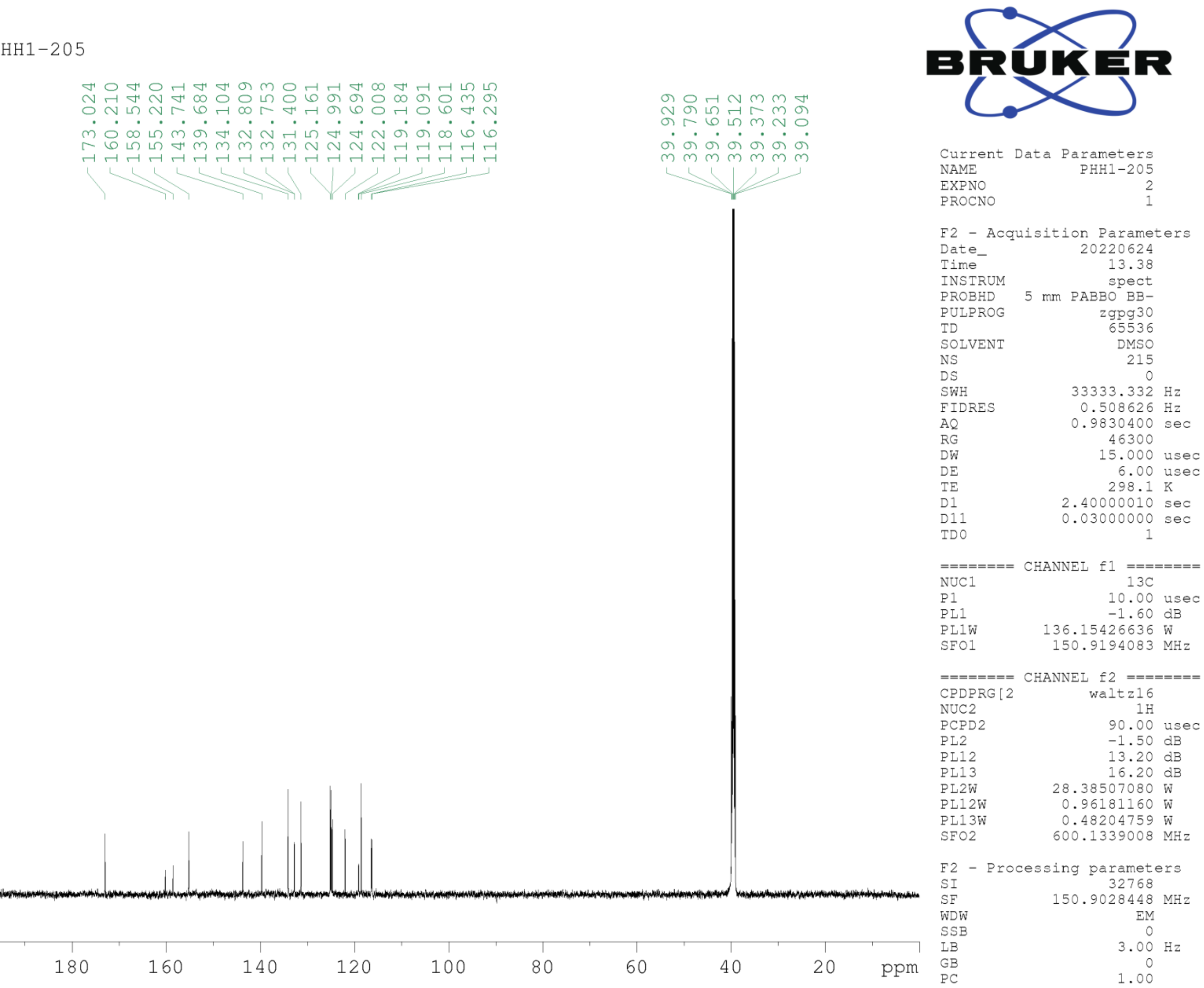

Figure S4. ^{13}C NMR (150 MHz, DMSO-d_6) for compound **6b**.

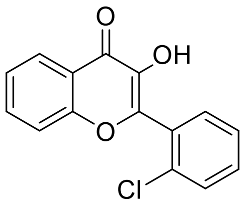

6c

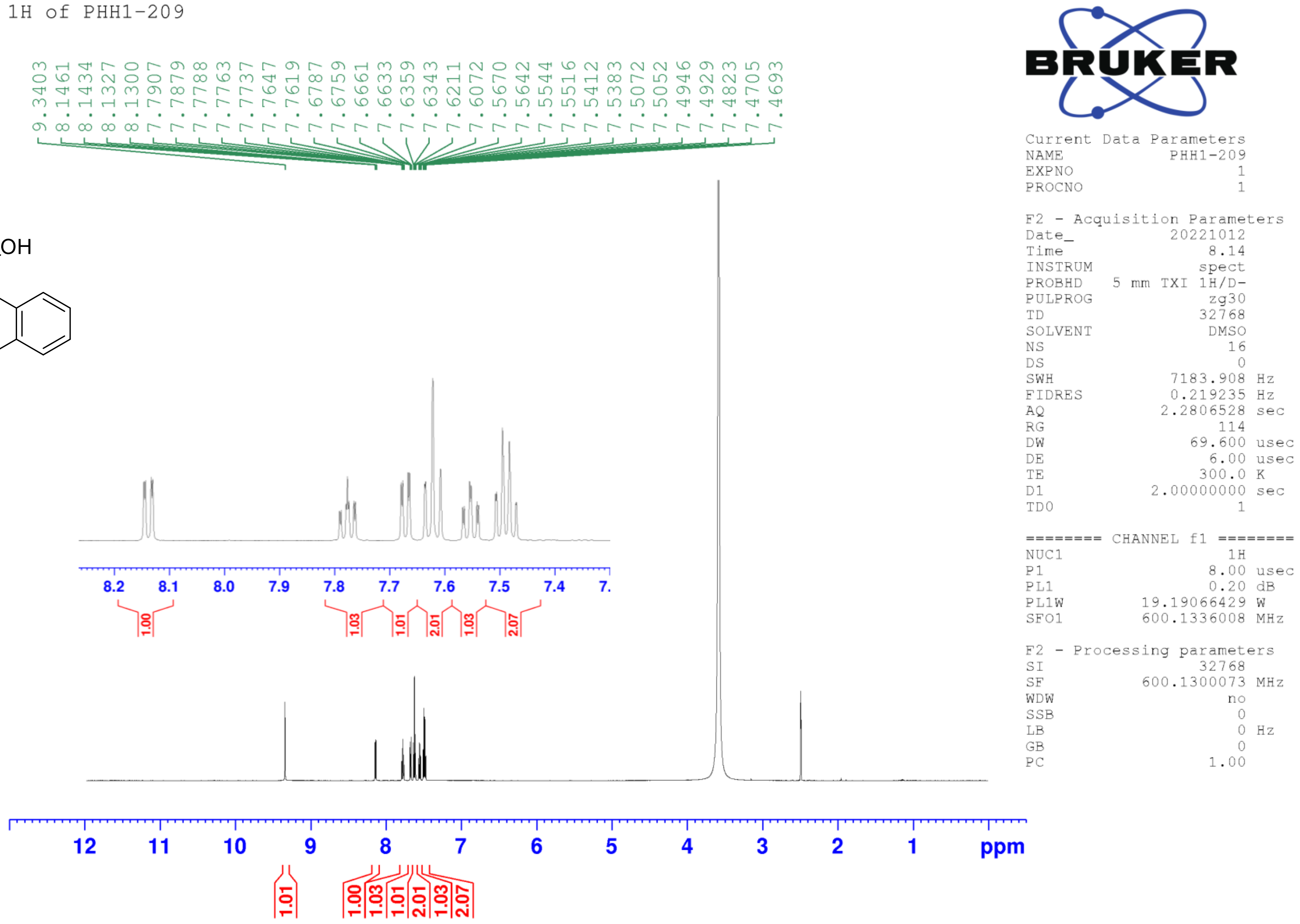

Figure S5. ^{1}H NMR (600 MHz, DMSO-d_6) for compound **6c**.

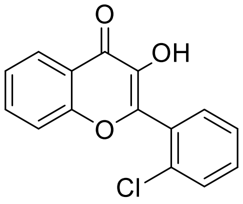

6c

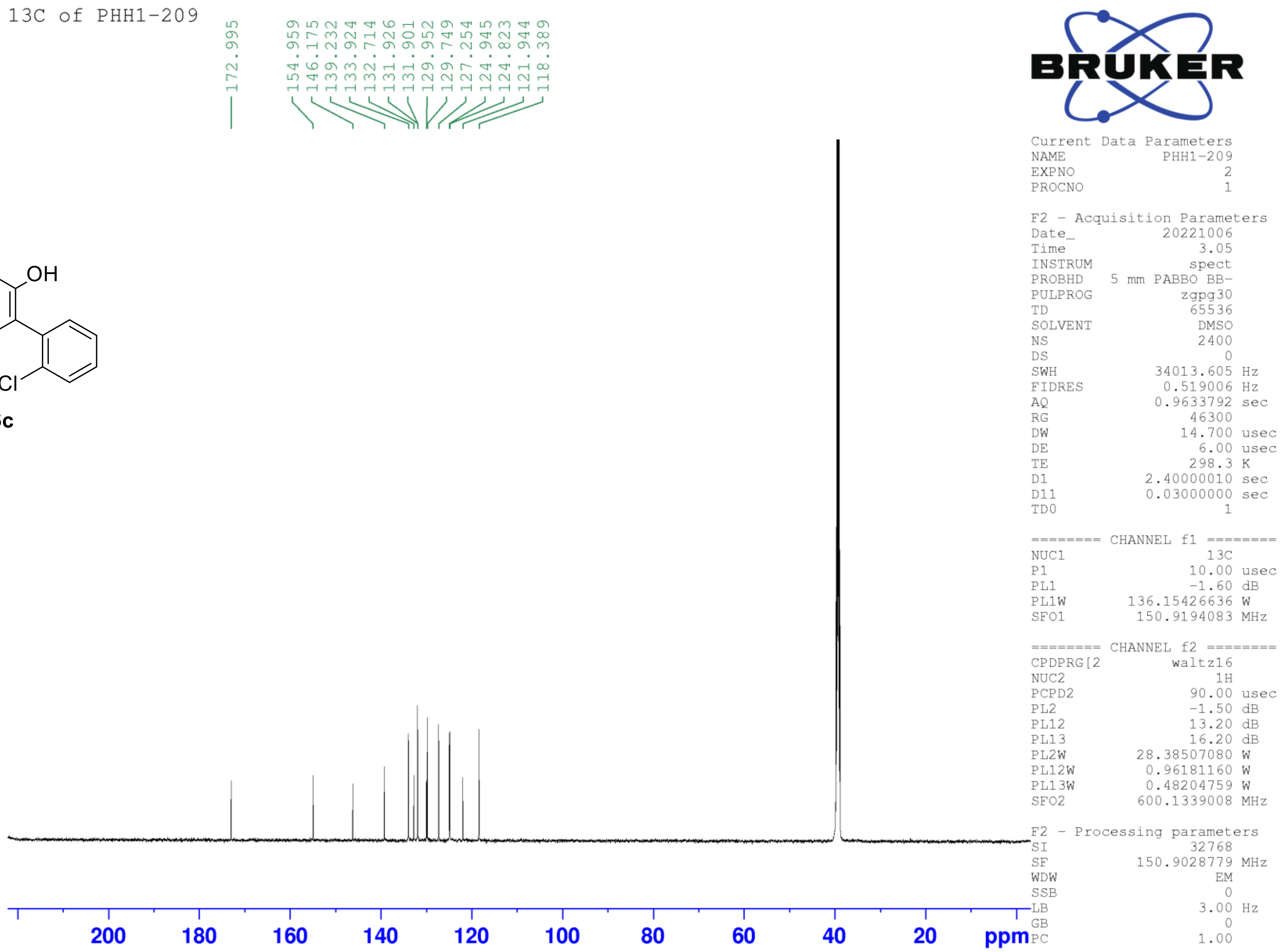

Figure S6. ^{13}C NMR (150 MHz, DMSO-d_6) for compound **6c**.

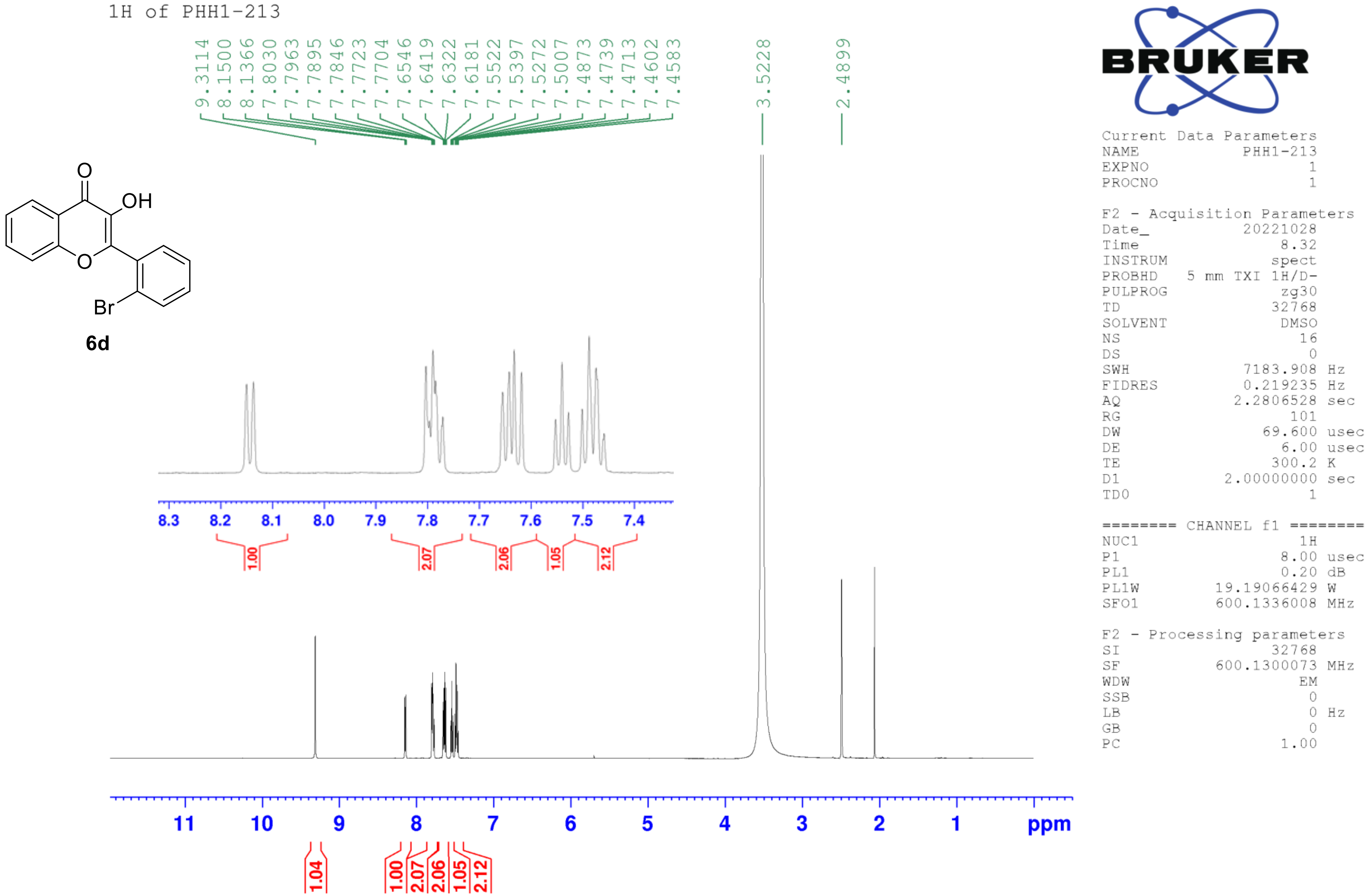

Figure S7. ^{1}H NMR (600 MHz, DMSO-d_6) for compound **6d**.

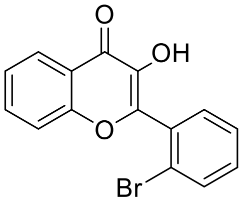

6d

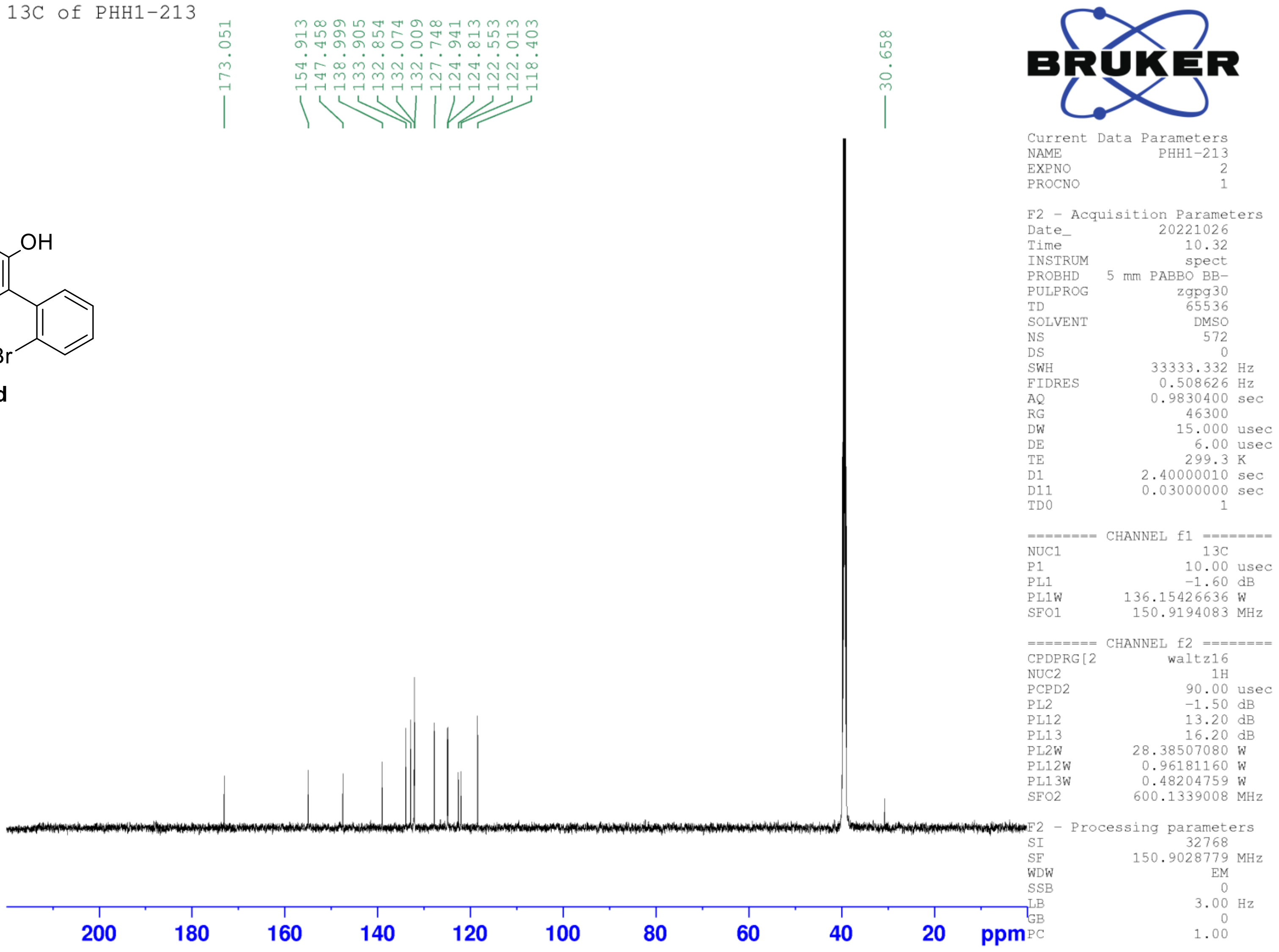

Figure S8. ^{13}C NMR (150 MHz, DMSO-d_6) for compound **6d**.

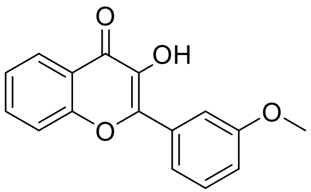

6e

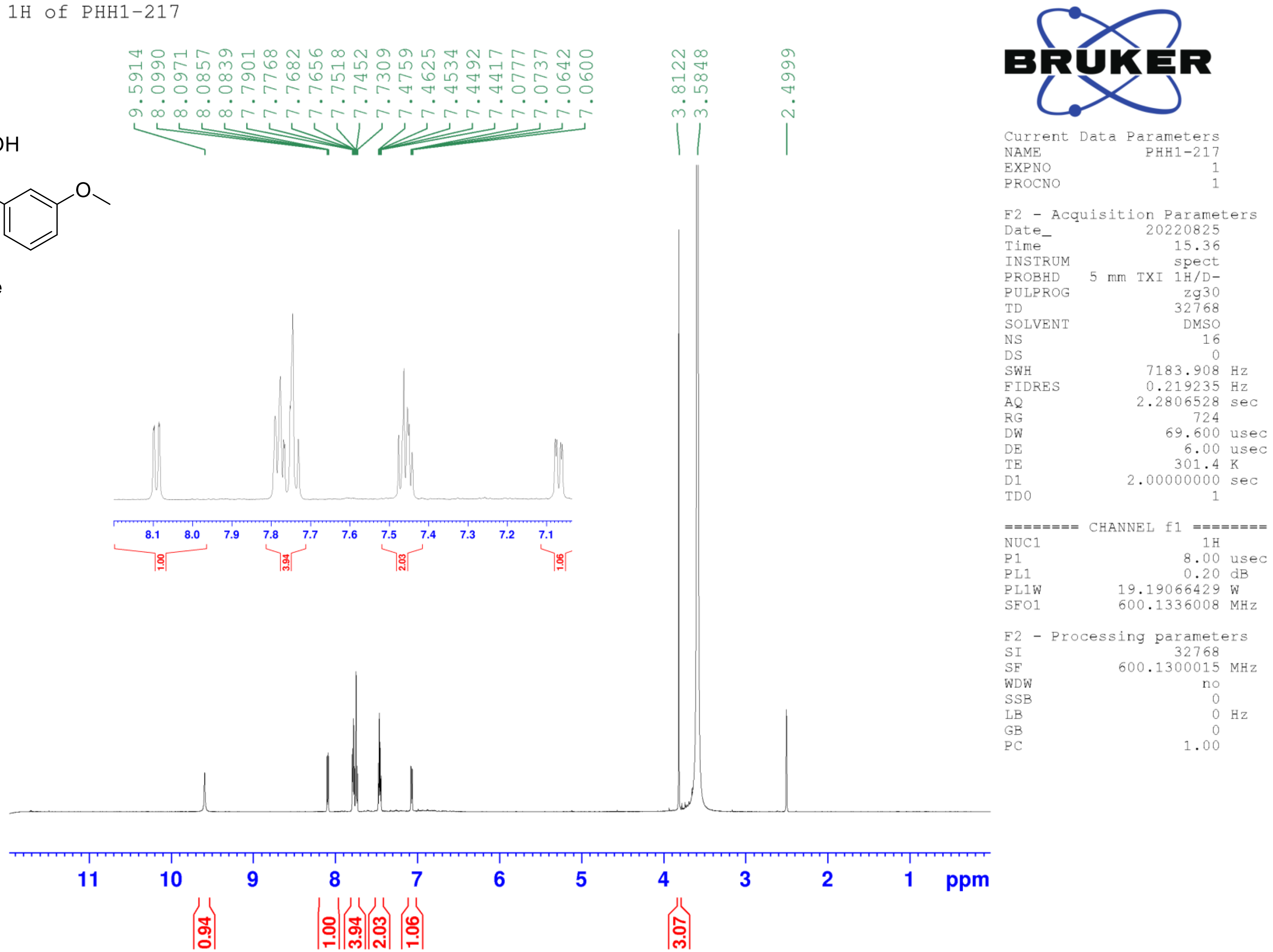

Figure S9. ^{1}H NMR (600 MHz, DMSO-d_6) for compound **6e**.

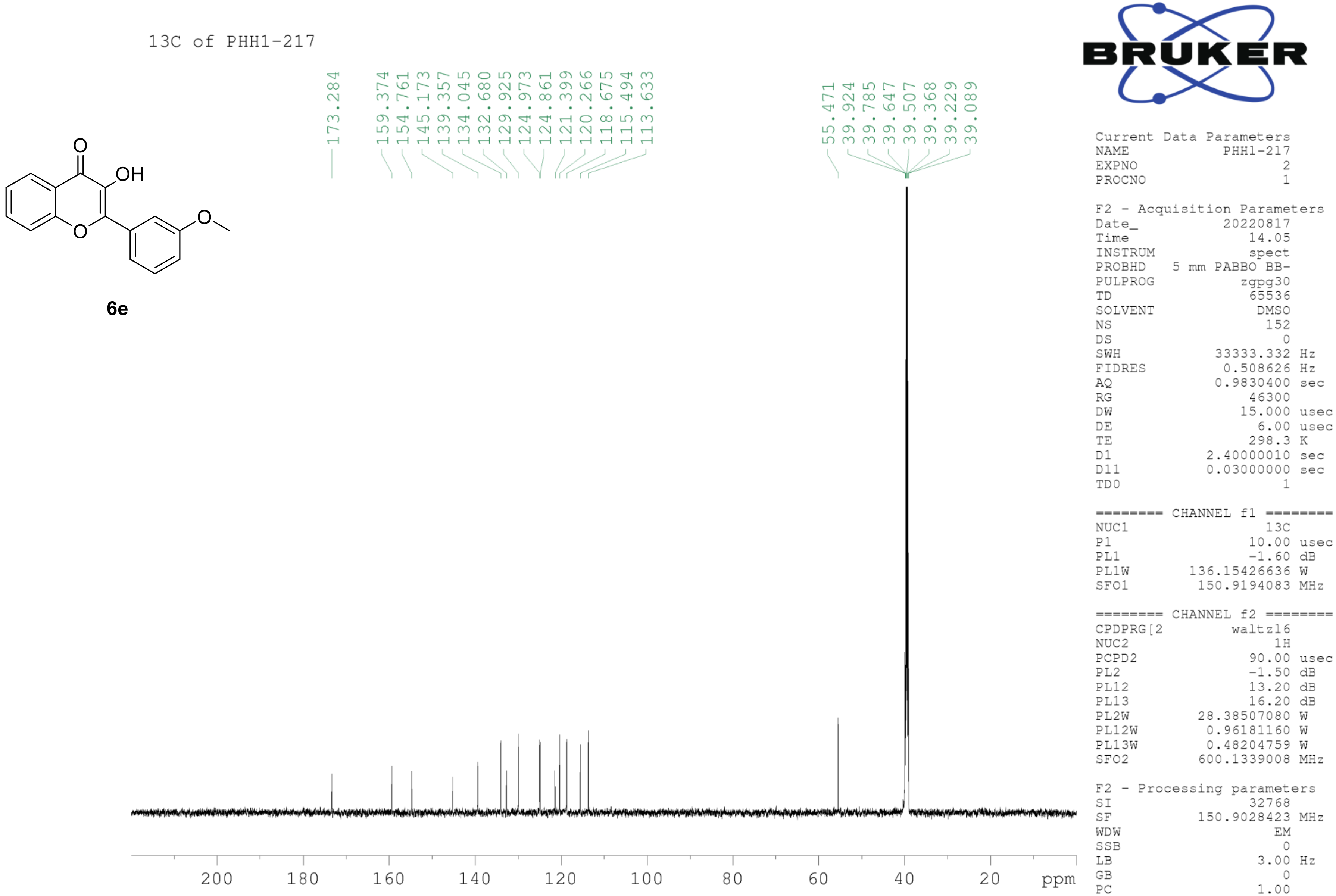

Figure S10. ^{13}C NMR (150 MHz, DMSO-d_6) for compound **6e**.

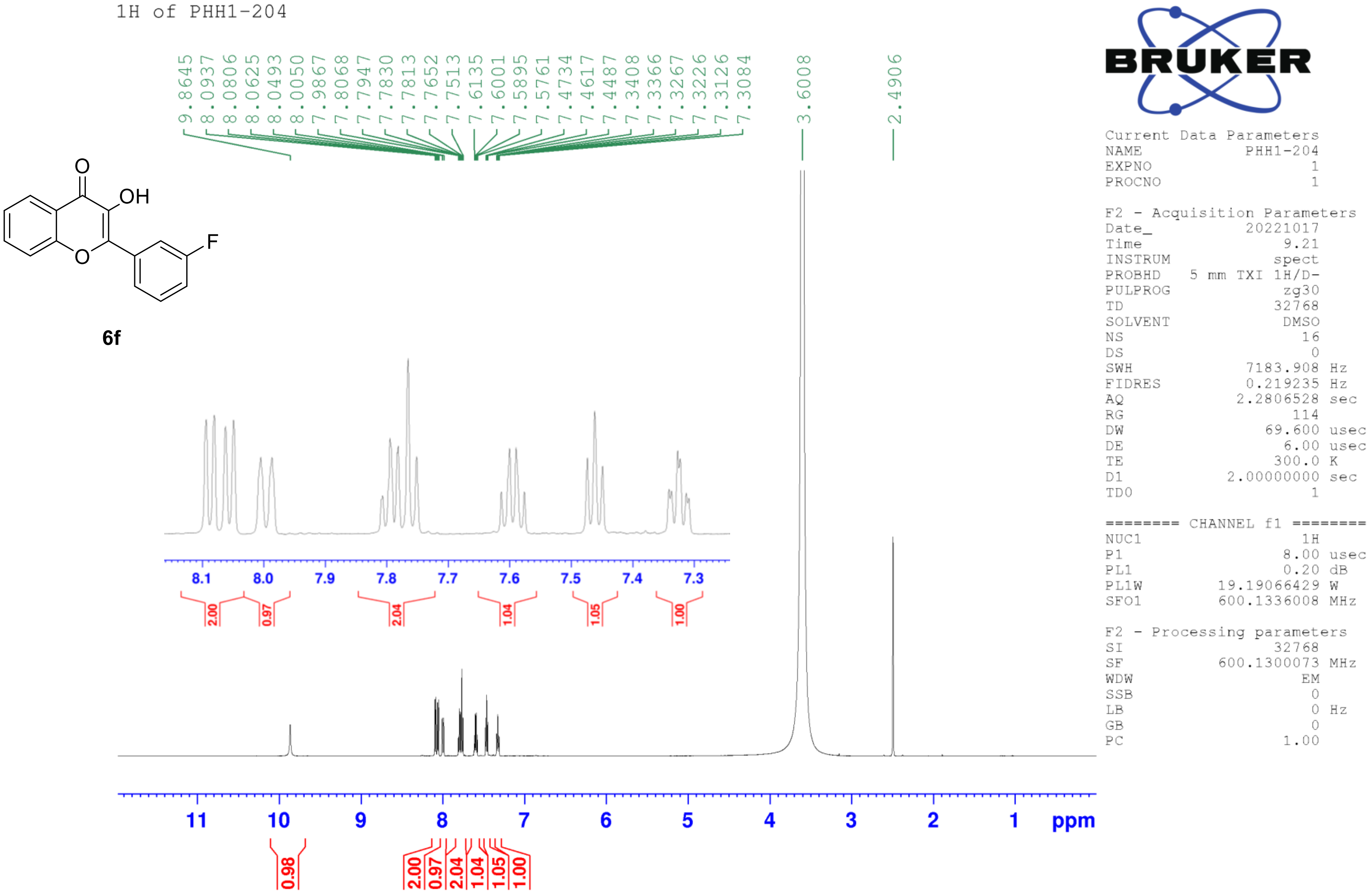

Figure S11. ^{1}H NMR (600 MHz, DMSO-d_6) for compound **6f**.

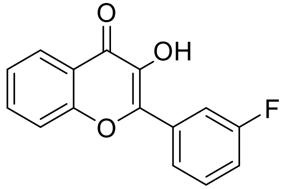

6f

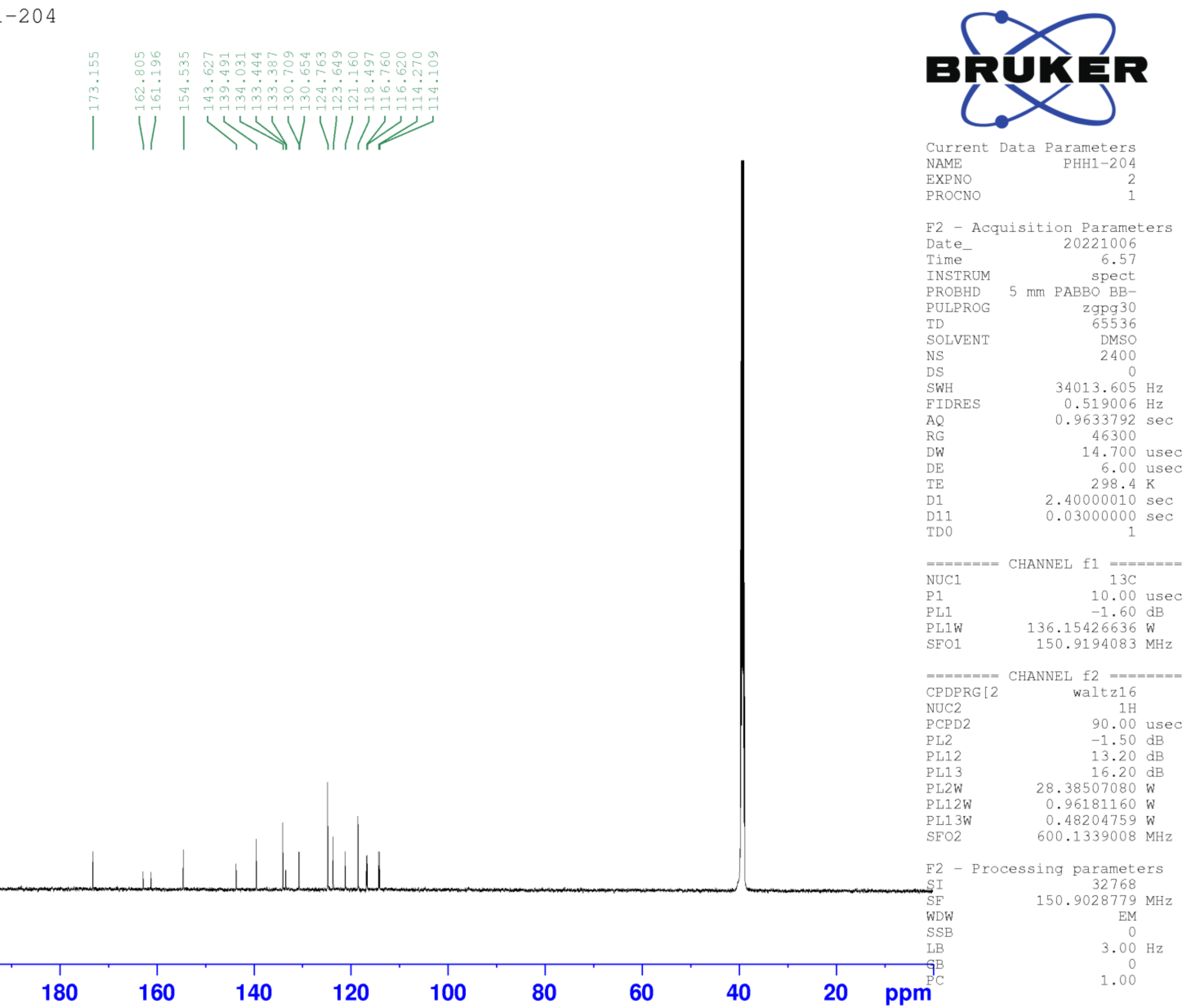

Figure S12. ^{13}C NMR (150 MHz, DMSO-d_6) for compound **6f**.

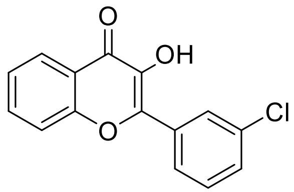

6g

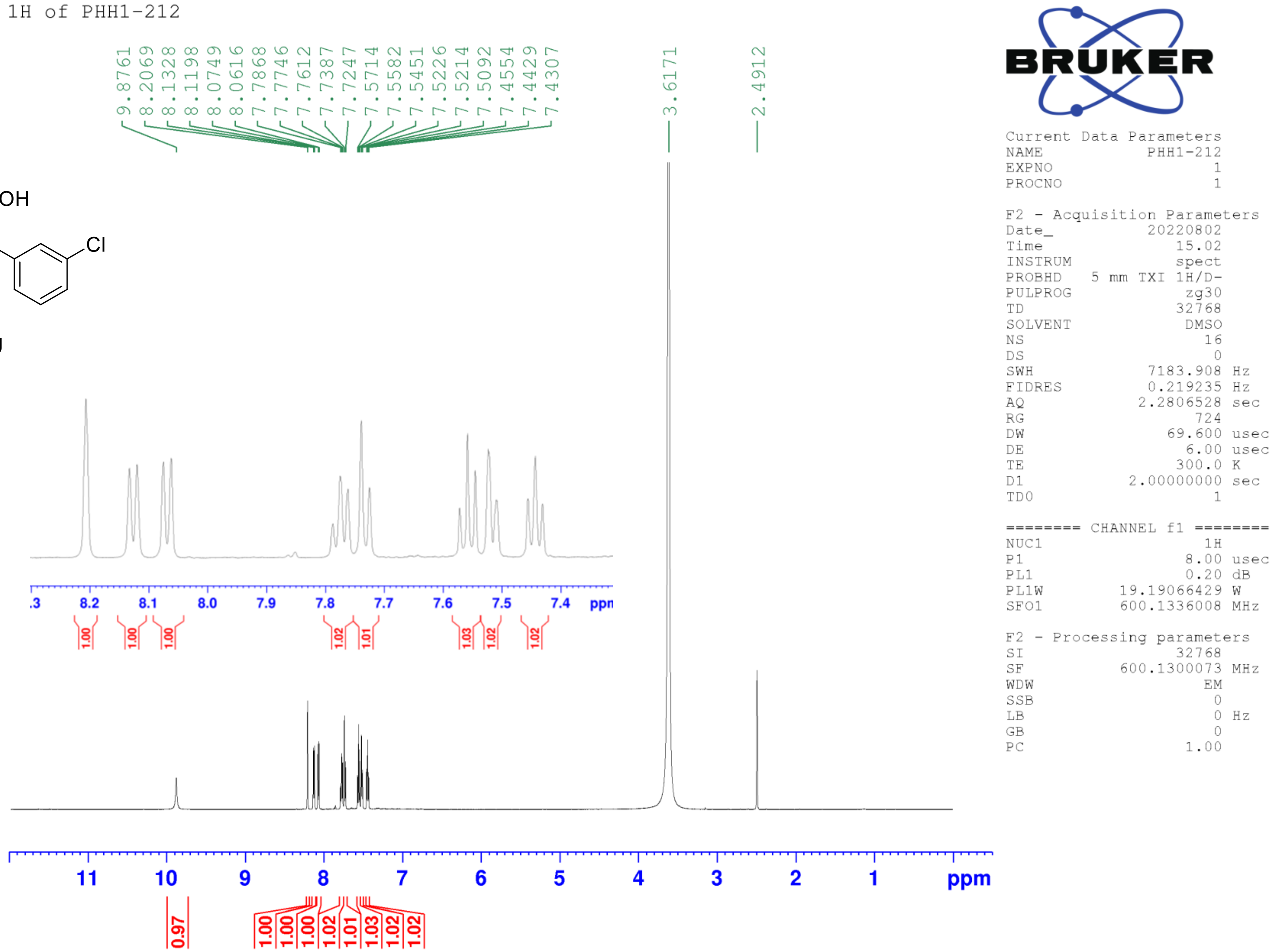

Figure S13. ^{1}H NMR (600 MHz, DMSO-d_6) for compound **6g**.

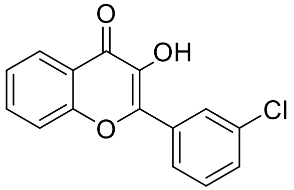

6g

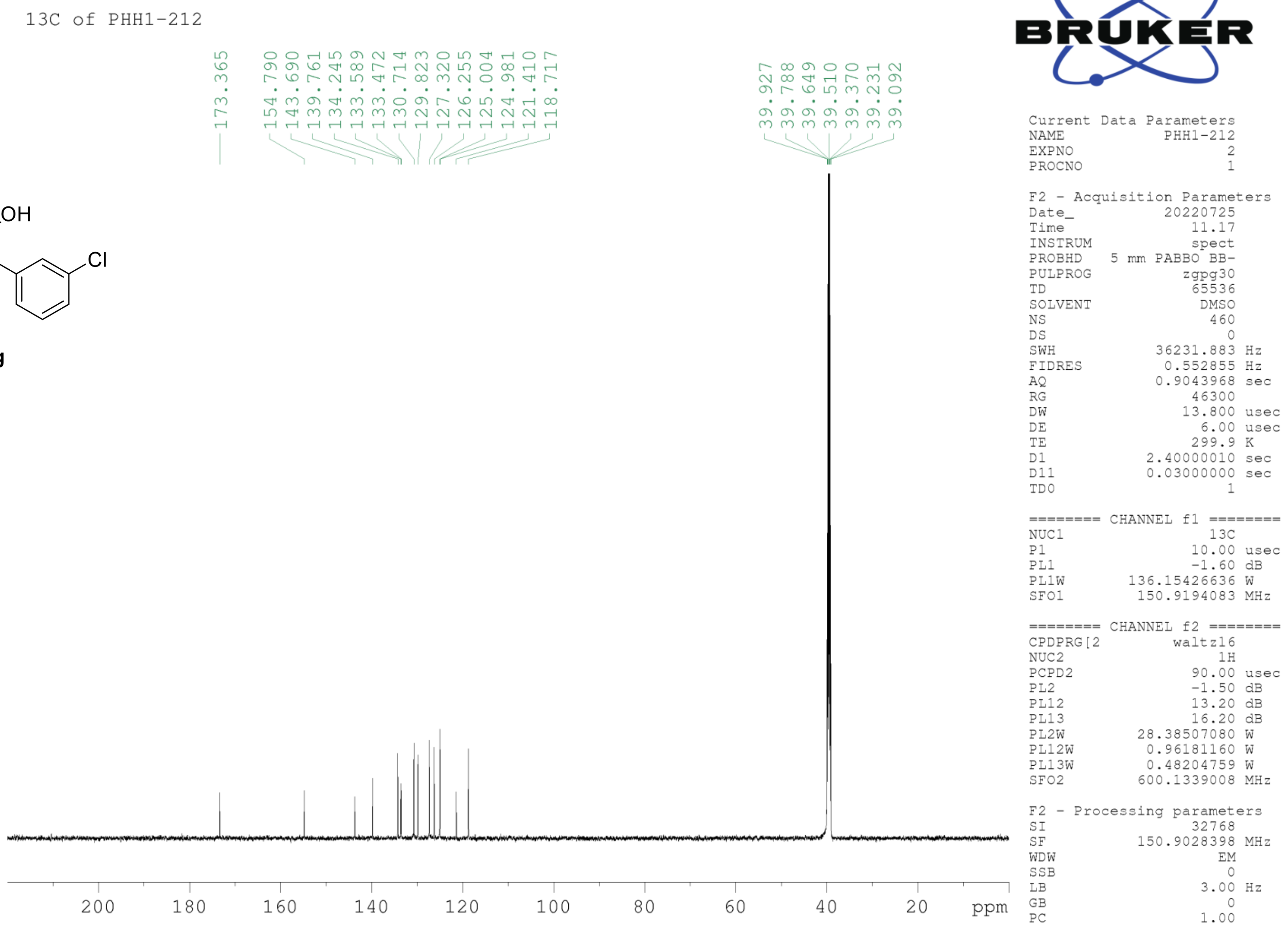

Figure S14. ^{13}C NMR (150 MHz, DMSO-d_6) for compound **6g**.

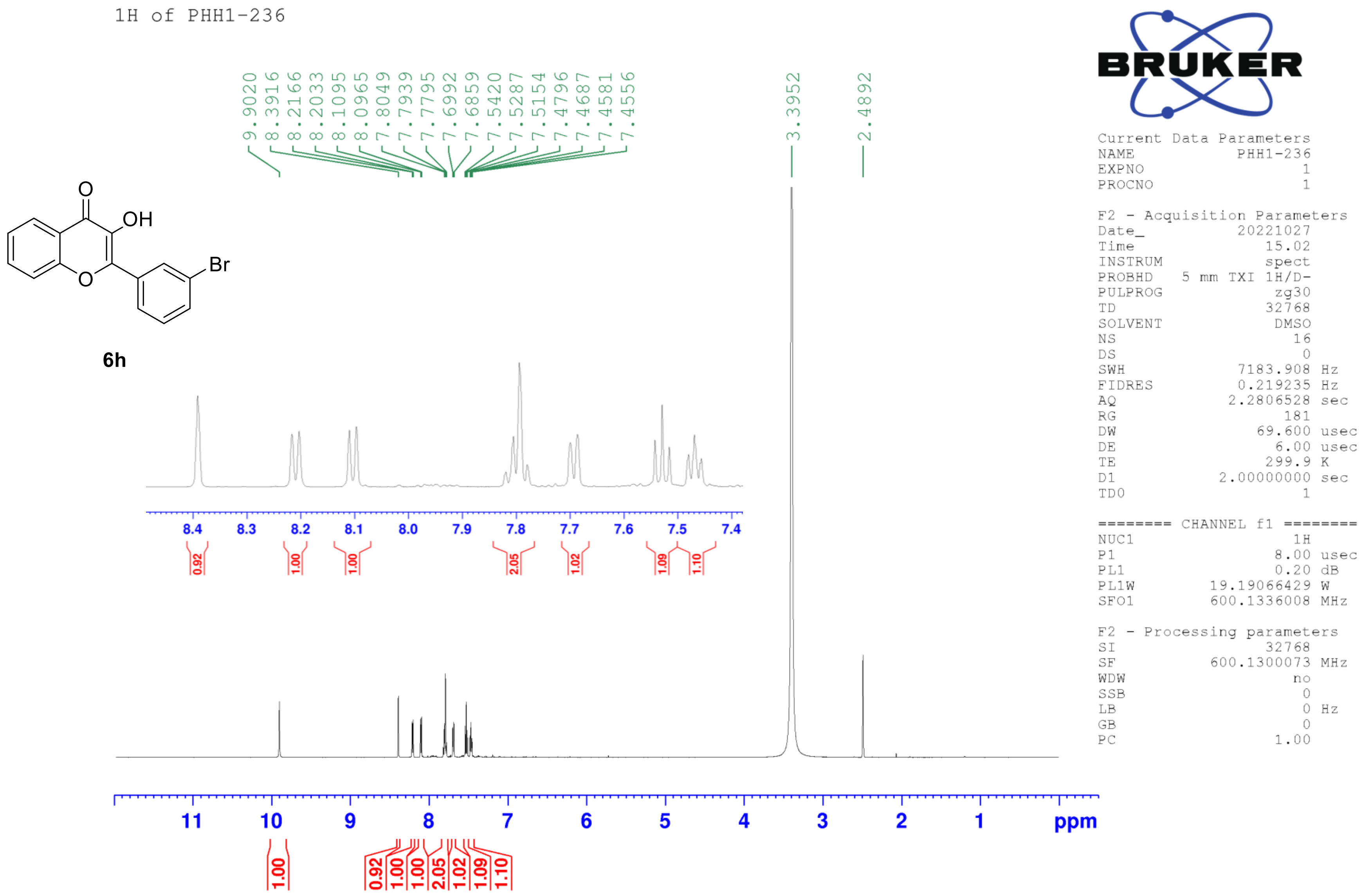

Figure S15. ^{1}H NMR (600 MHz, DMSO-d_6) for compound **6h**.

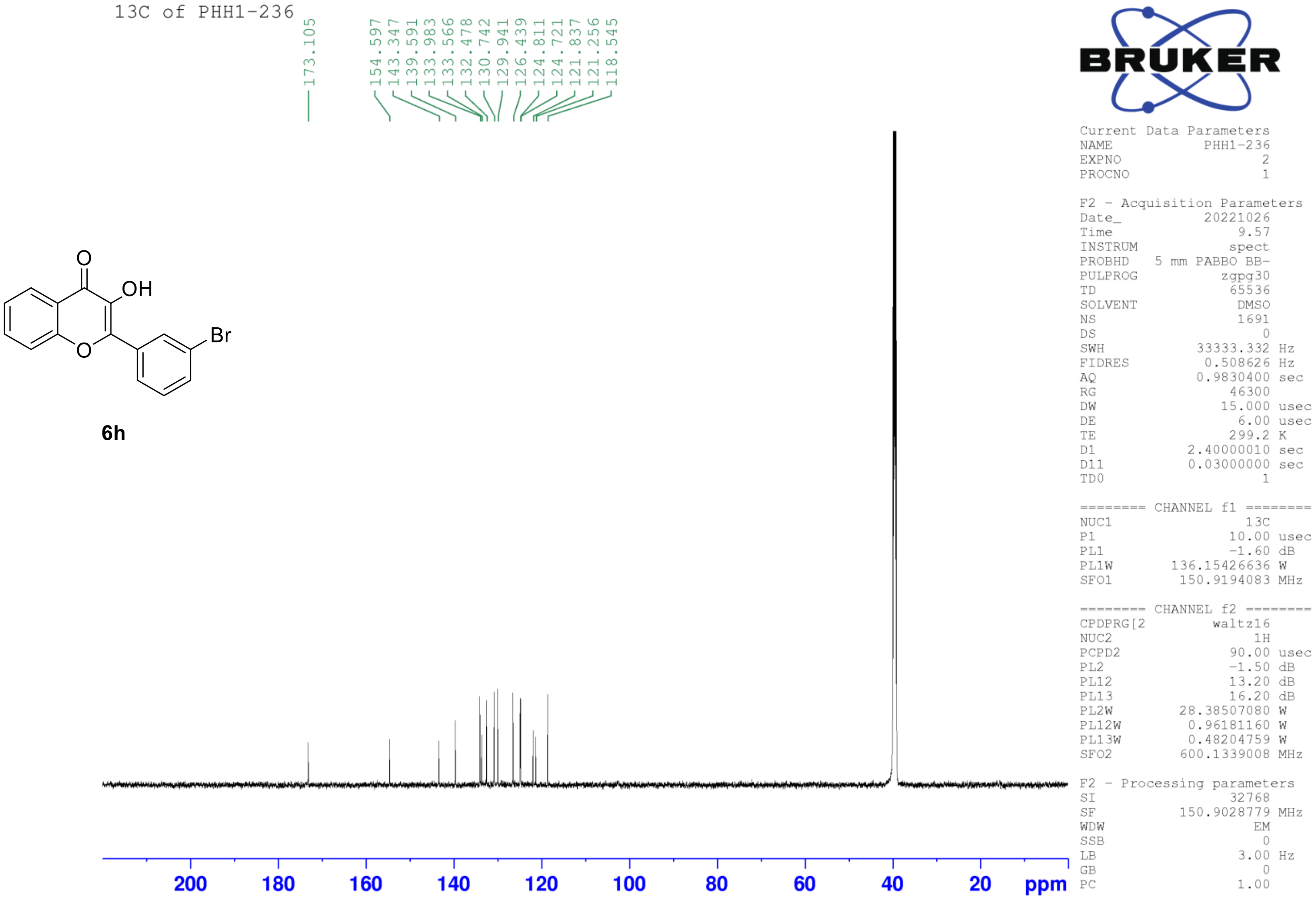

Figure S16. ^{13}C NMR (150 MHz, DMSO-d_6) for compound **6h**.

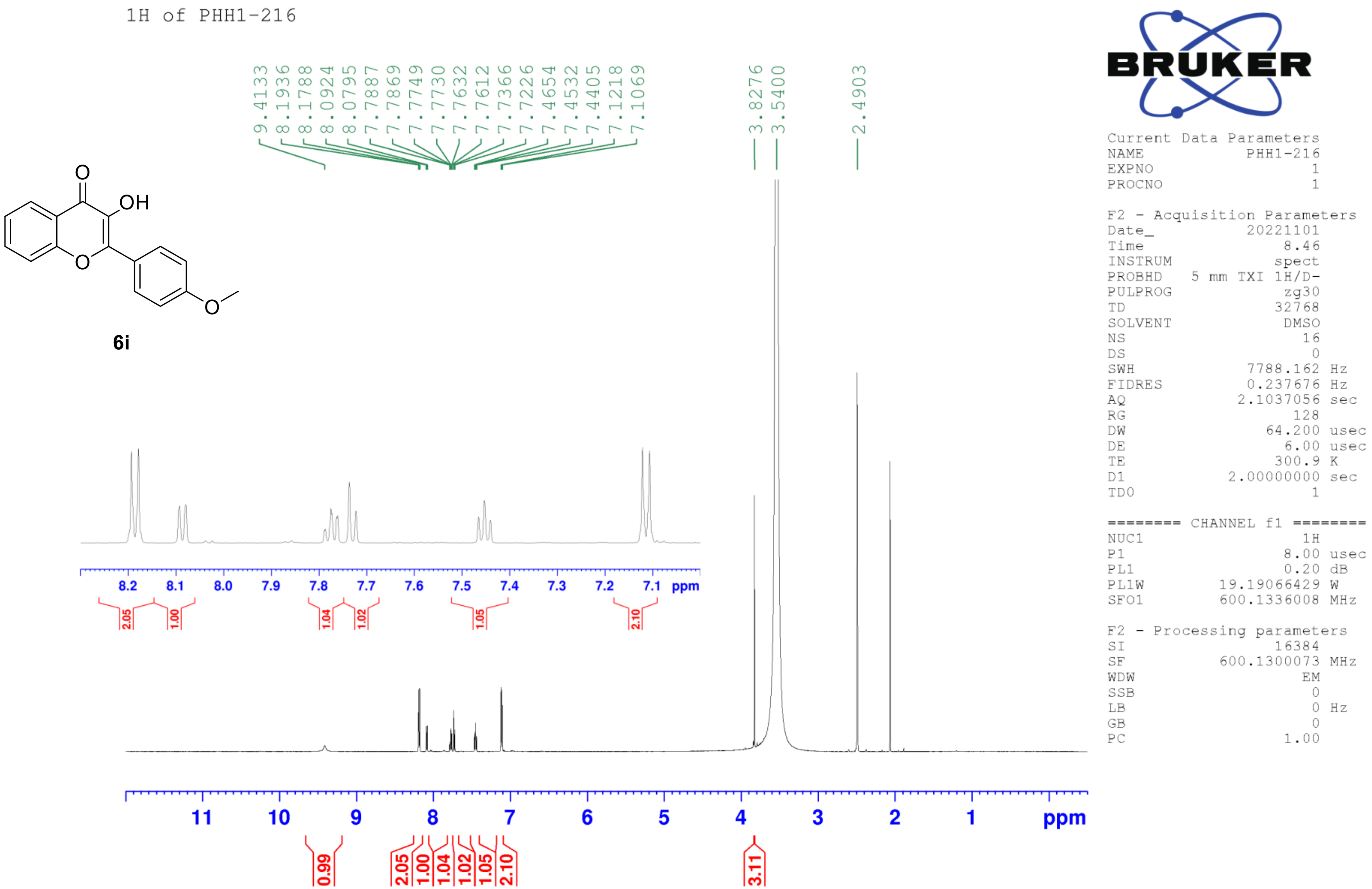

Figure S17. ^{1}H NMR (600 MHz, DMSO-d_6) for compound **6i**.

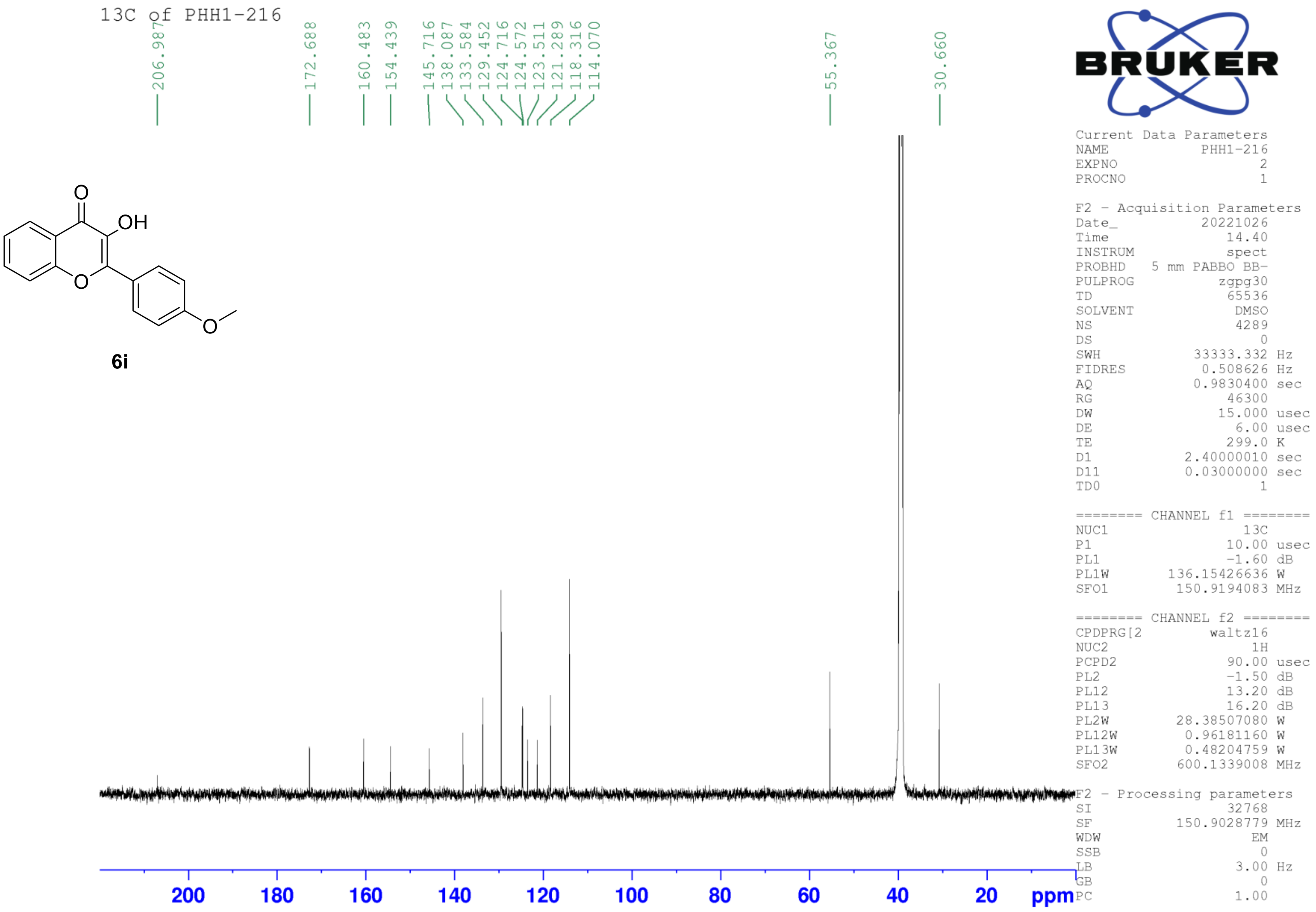

Figure S18. ^{13}C NMR (150 MHz, DMSO-d_6) for compound **6i**.

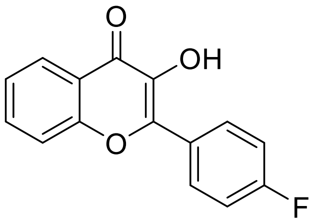

6j

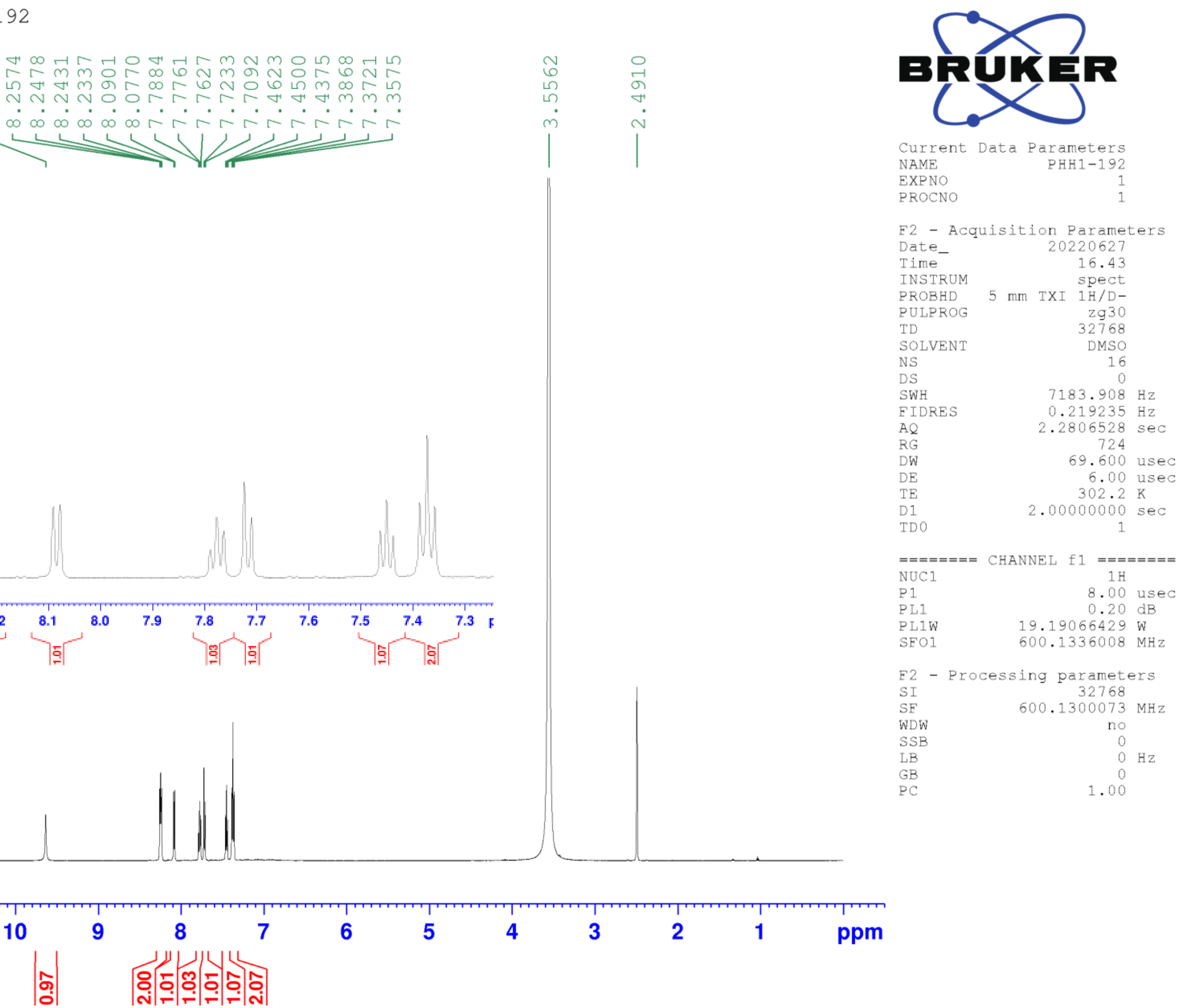

Figure S19. ^{1}H NMR (600 MHz, DMSO-d_6) for compound **6j**.

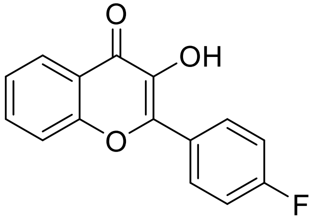

6j

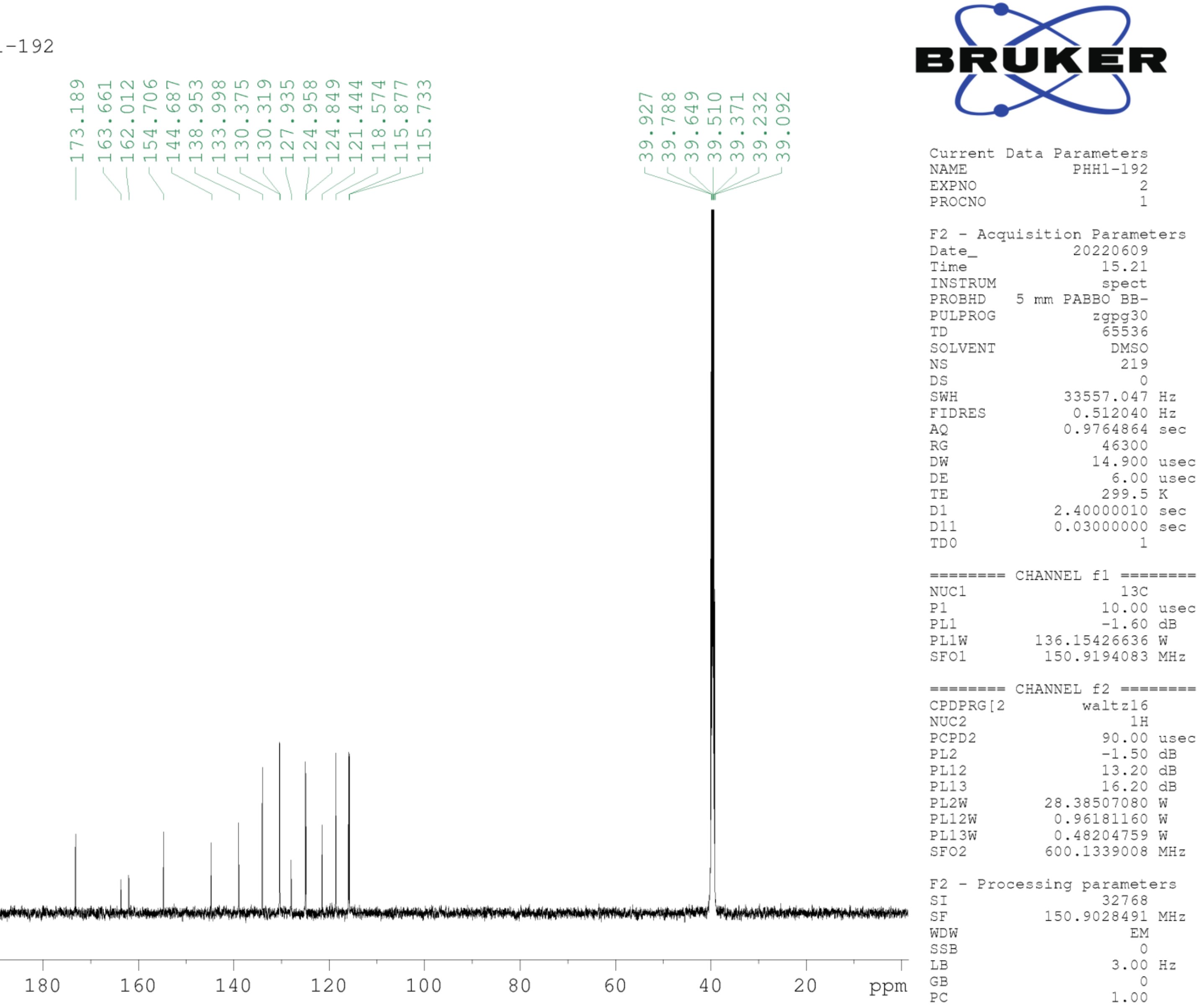

Figure S20. ^{13}C NMR (150 MHz, DMSO-d_6) for compound **6j**.

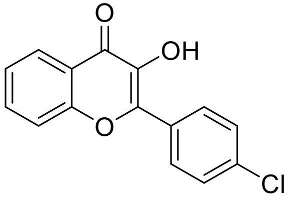

6k

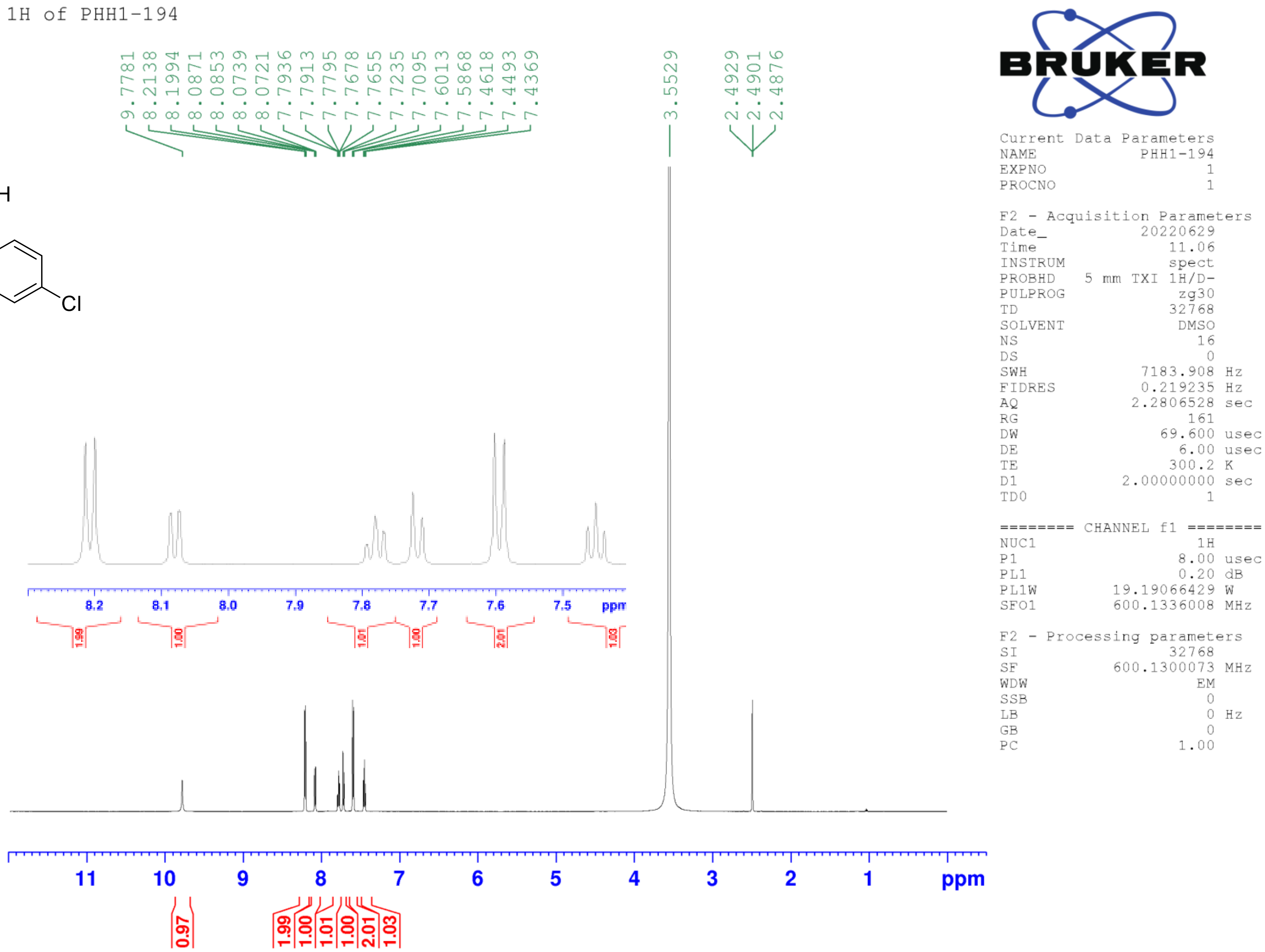

Figure S21. ^{1}H NMR (600 MHz, DMSO-d_6) for compound **6k**.

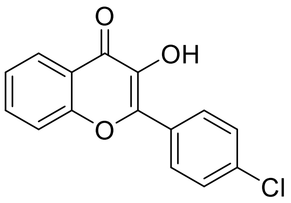

6k

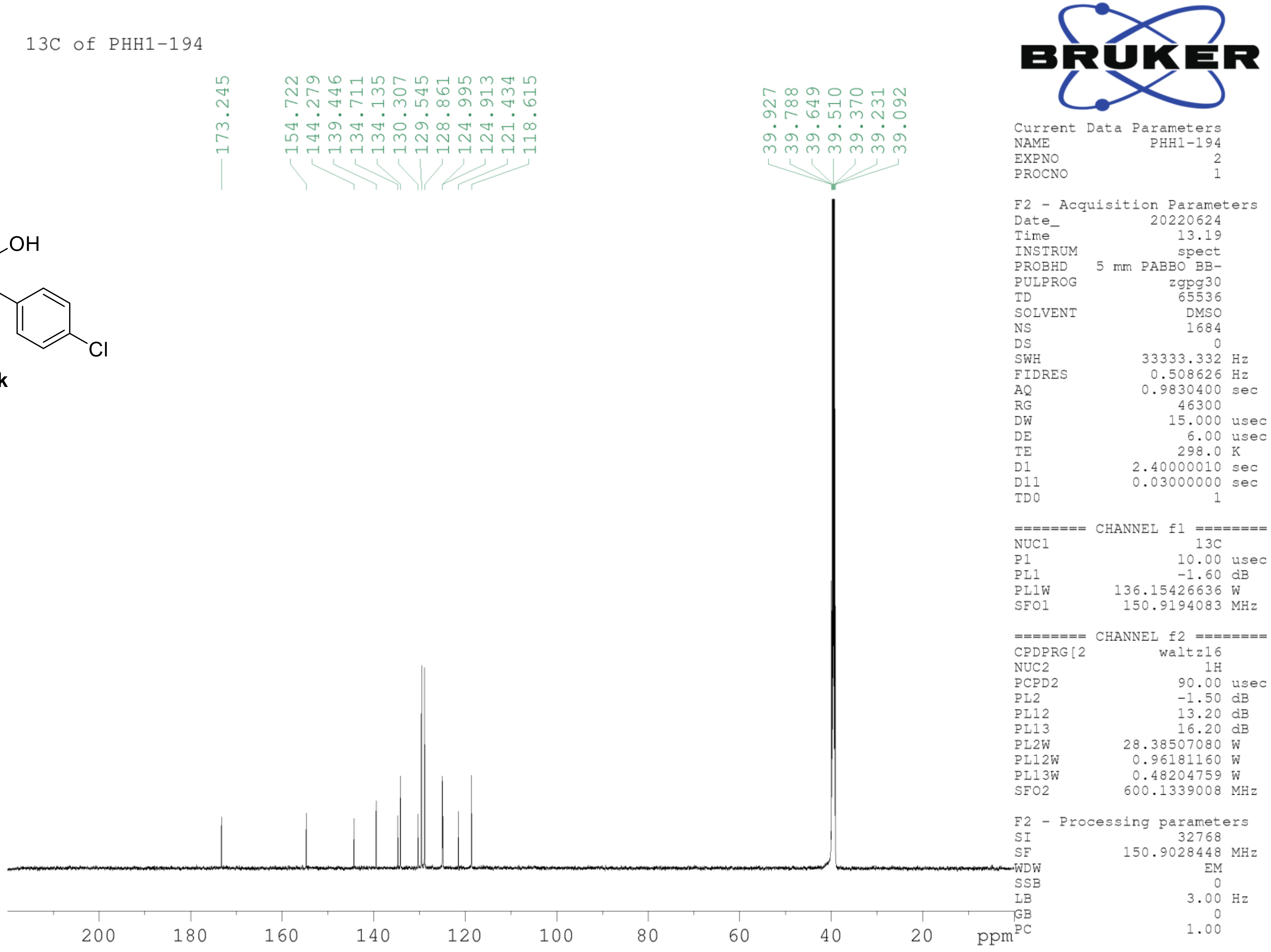

Figure S22. ^{13}C NMR (150 MHz, DMSO-d_6) for compound **6k**.

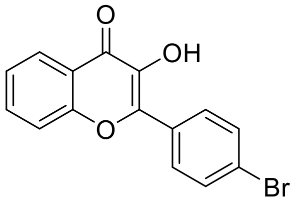

6l

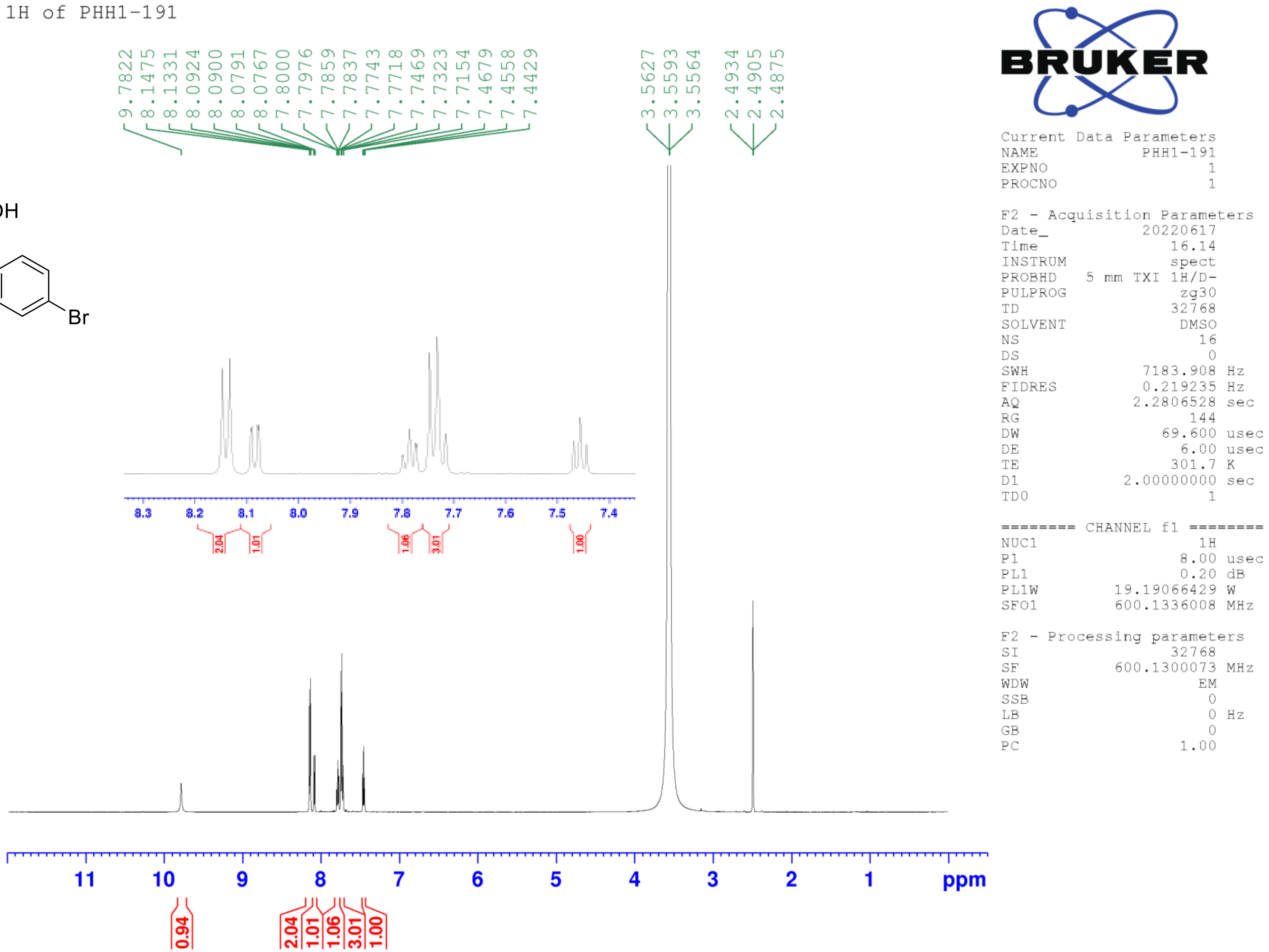

Figure S23. ^{1}H NMR (600 MHz, DMSO-d_6) for compound **6l**.

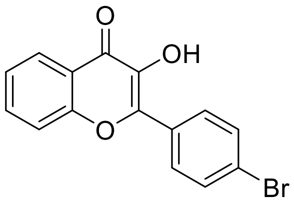

6l

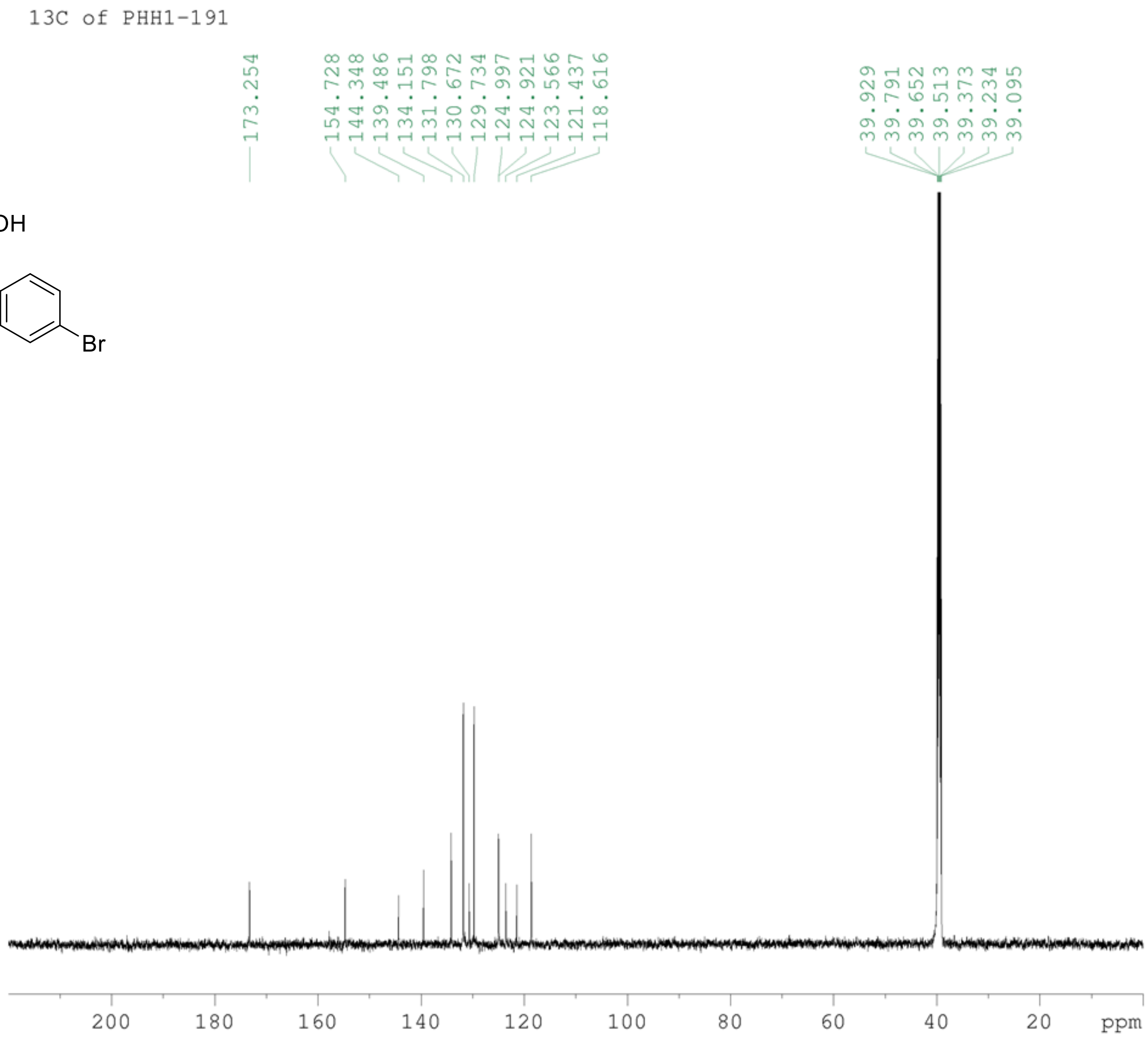


```
Current Data Parameters
NAME              PHH1-191
EXPNO                    2
PROCNO                   1

F2 - Acquisition Parameters
Date_             20220609
Time                 15.06
INSTRUM              spect
PROBHD    5 mm PABBO BB-
PULPROG            zgpg30
TD                  65536
SOLVENT              DMSO
NS                    353
DS                      0
SWH             33557.047 Hz
FIDRES           0.512040 Hz
AQ              0.9764864 sec
RG                  46300
DW                 14.900 usec
DE                   6.00 usec
TE                  299.6 K
D1             2.40000010 sec
D11            0.03000000 sec
TD0                     1

======== CHANNEL f1 ========
NUC1                  13C
P1                  10.00 usec
PL1                 -1.60 dB
PL1W         136.15426636 W
SFO1          150.9194083 MHz

======== CHANNEL f2 ========
CPDPRG[2          waltz16
NUC2                   1H
PCPD2               90.00 usec
PL2                 -1.50 dB
PL12                13.20 dB
PL13                16.20 dB
PL2W          28.38507080 W
PL12W          0.96181160 W
PL13W          0.48204759 W
SFO2          600.1339008 MHz

F2 - Processing parameters
SI                  32768
SF            150.9028466 MHz
WDW                    EM
SSB                     0
LB                   3.00 Hz
GB                      0
PC                   1.00
```

Figure S24. ^{13}C NMR (150 MHz, DMSO-d_6) for compound **6l**.

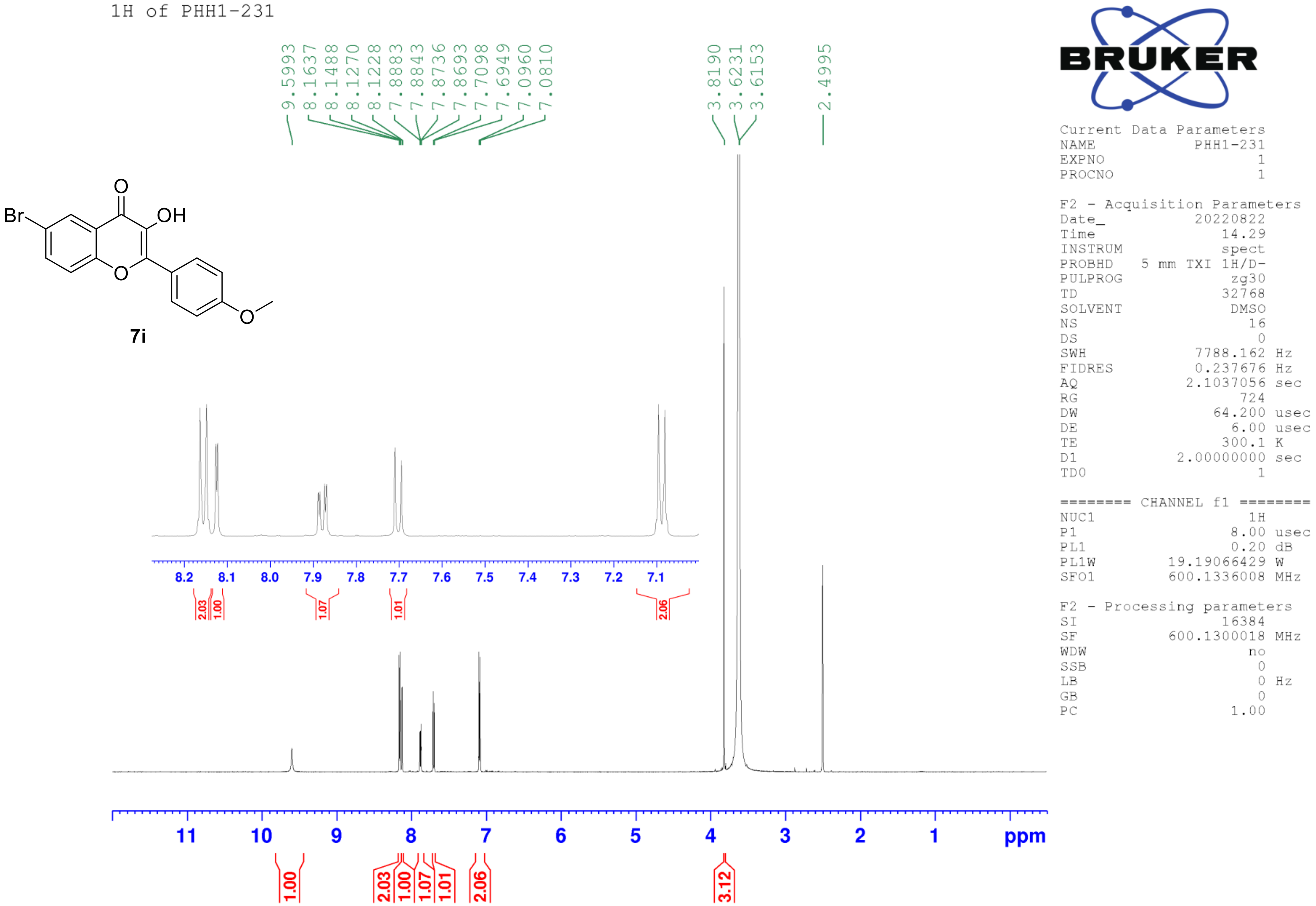

Figure S25. ^{1}H NMR (600 MHz, DMSO-d_6) for compound **7i**.

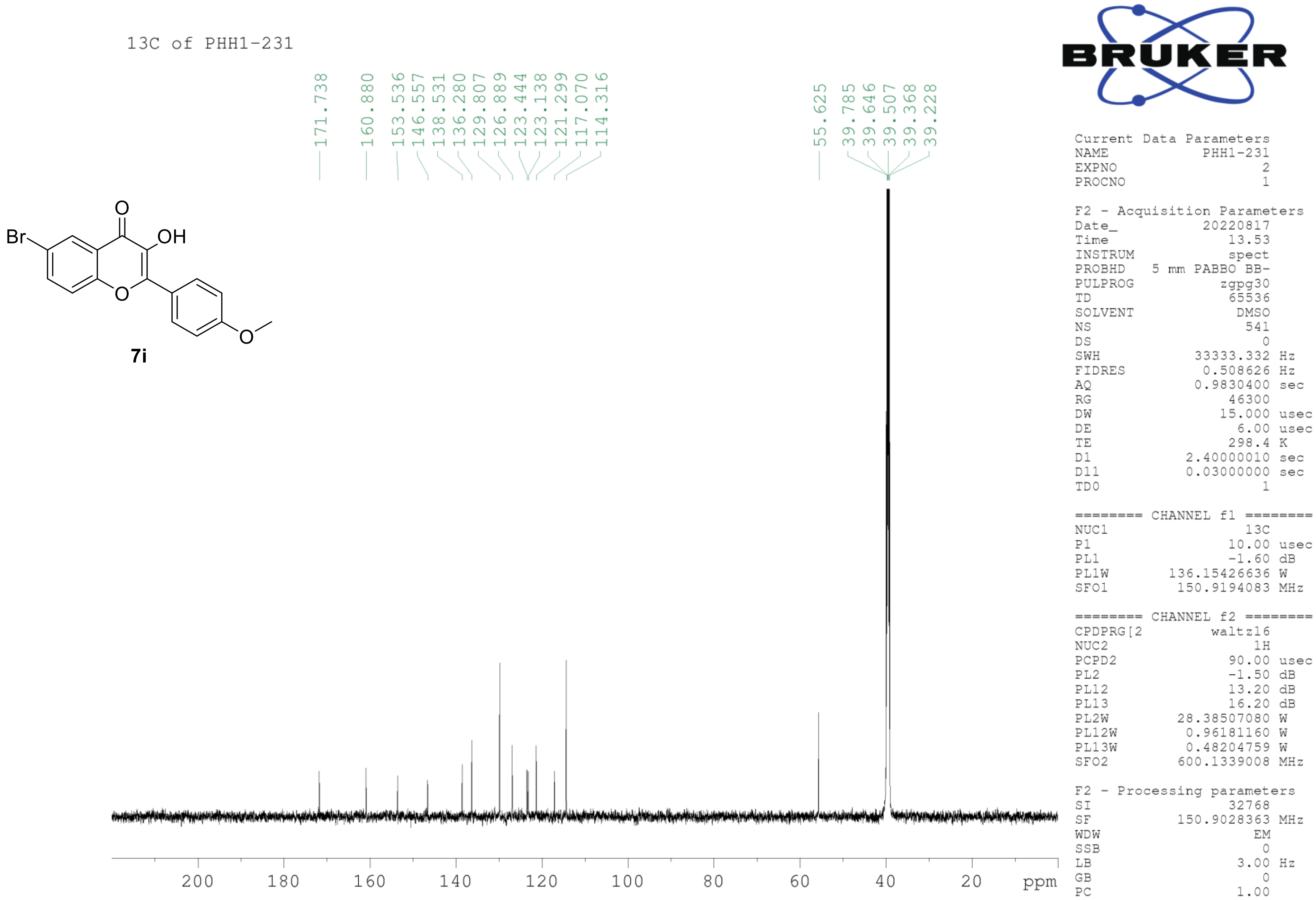

Figure S26. ^{13}C NMR (150 MHz, DMSO-d_6) for compound **7i**.

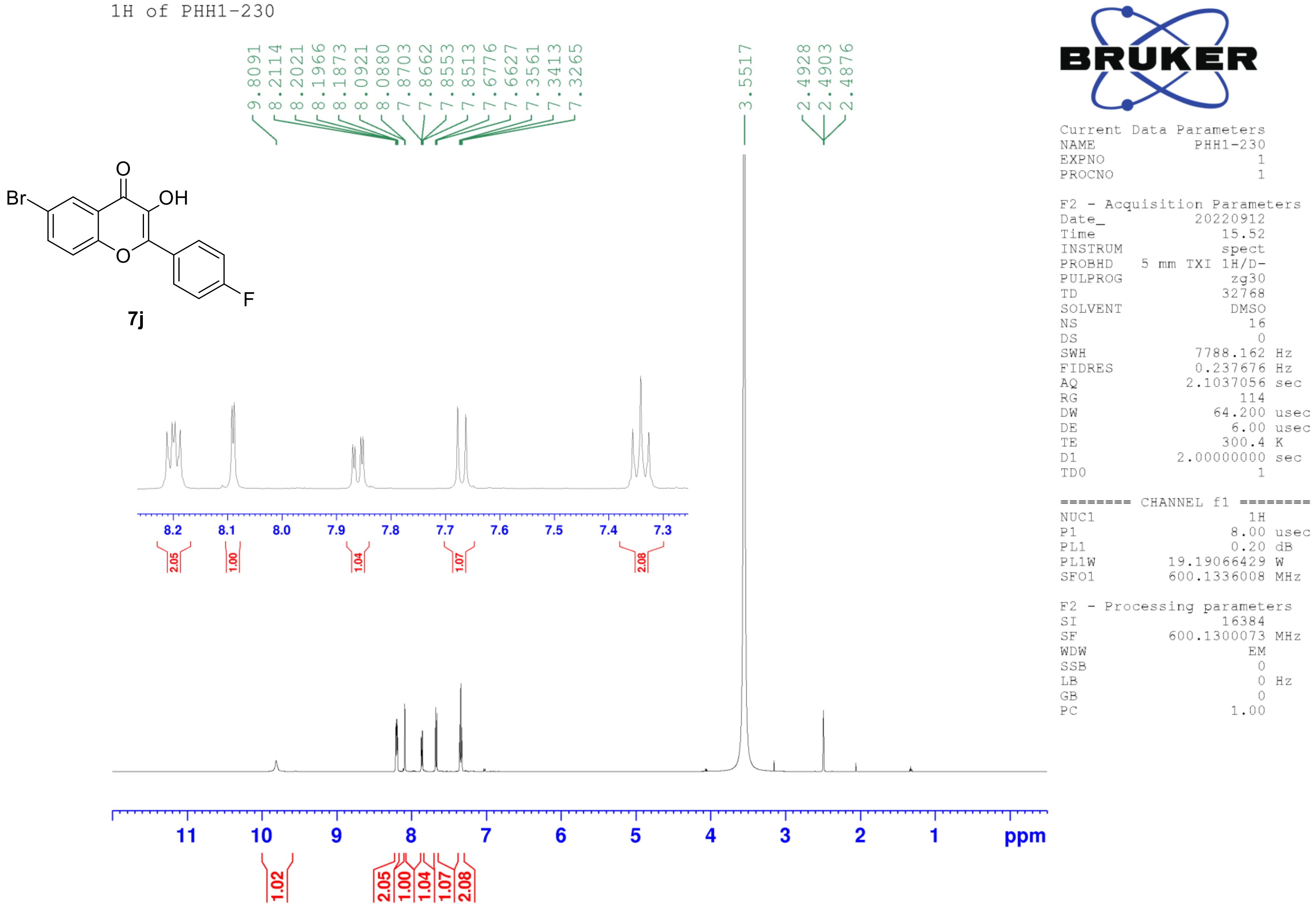

Figure S27. ^{1}H NMR (600 MHz, DMSO-d_6) for compound **7j**.

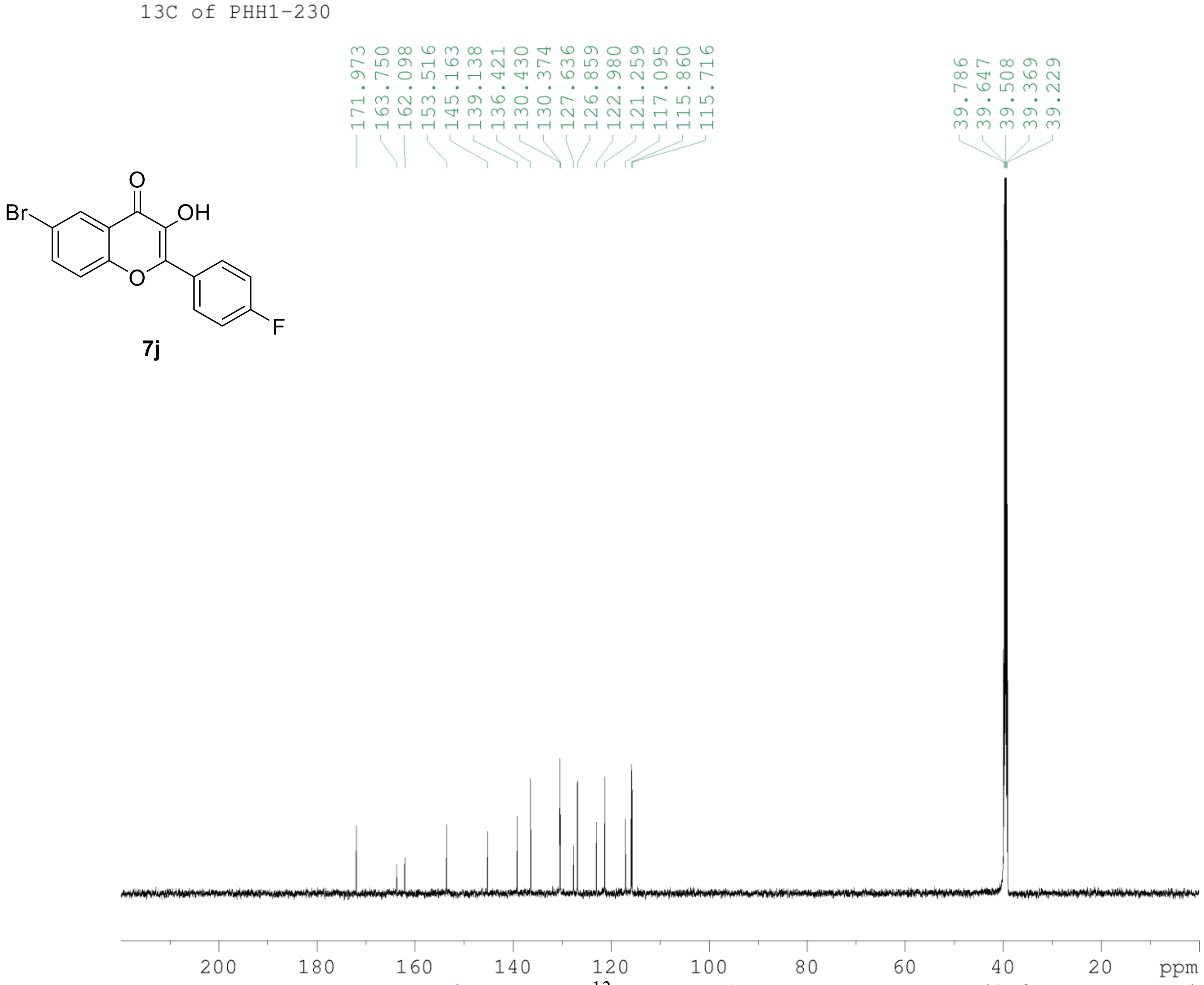

Current Data Parameters
NAME PHH1-230
EXPNO 2
PROCNO 1

F2 - Acquisition Parameters
Date_ 20220908
Time 16.02
INSTRUM spect
PROBHD 5 mm PABBO BB-
PULPROG zgpg30
TD 65536
SOLVENT DMSO
NS 248
DS 0
SWH 33333.332 Hz
FIDRES 0.508626 Hz
AQ 0.9830400 sec
RG 46300
DW 15.000 usec
DE 6.00 usec
TE 299.4 K
D1 2.40000010 sec
D11 0.03000000 sec
TD0 1

======== CHANNEL f1 ========
NUC1 13C
P1 10.00 usec
PL1 -1.60 dB
PL1W 136.15426636 W
SFO1 150.9194083 MHz

======== CHANNEL f2 ========
CPDPRG[2 waltz16
NUC2 1H
PCPD2 90.00 usec
PL2 -1.50 dB
PL12 13.20 dB
PL13 16.20 dB
PL2W 28.38507080 W
PL12W 0.96181160 W
PL13W 0.48204759 W
SFO2 600.1339008 MHz

F2 - Processing parameters
SI 32768
SF 150.9028466 MHz
WDW EM
SSB 0
LB 3.00 Hz
GB 0
PC 1.00

Figure S28. ^{13}C NMR (150 MHz, DMSO-d_6) for compound **7j**.

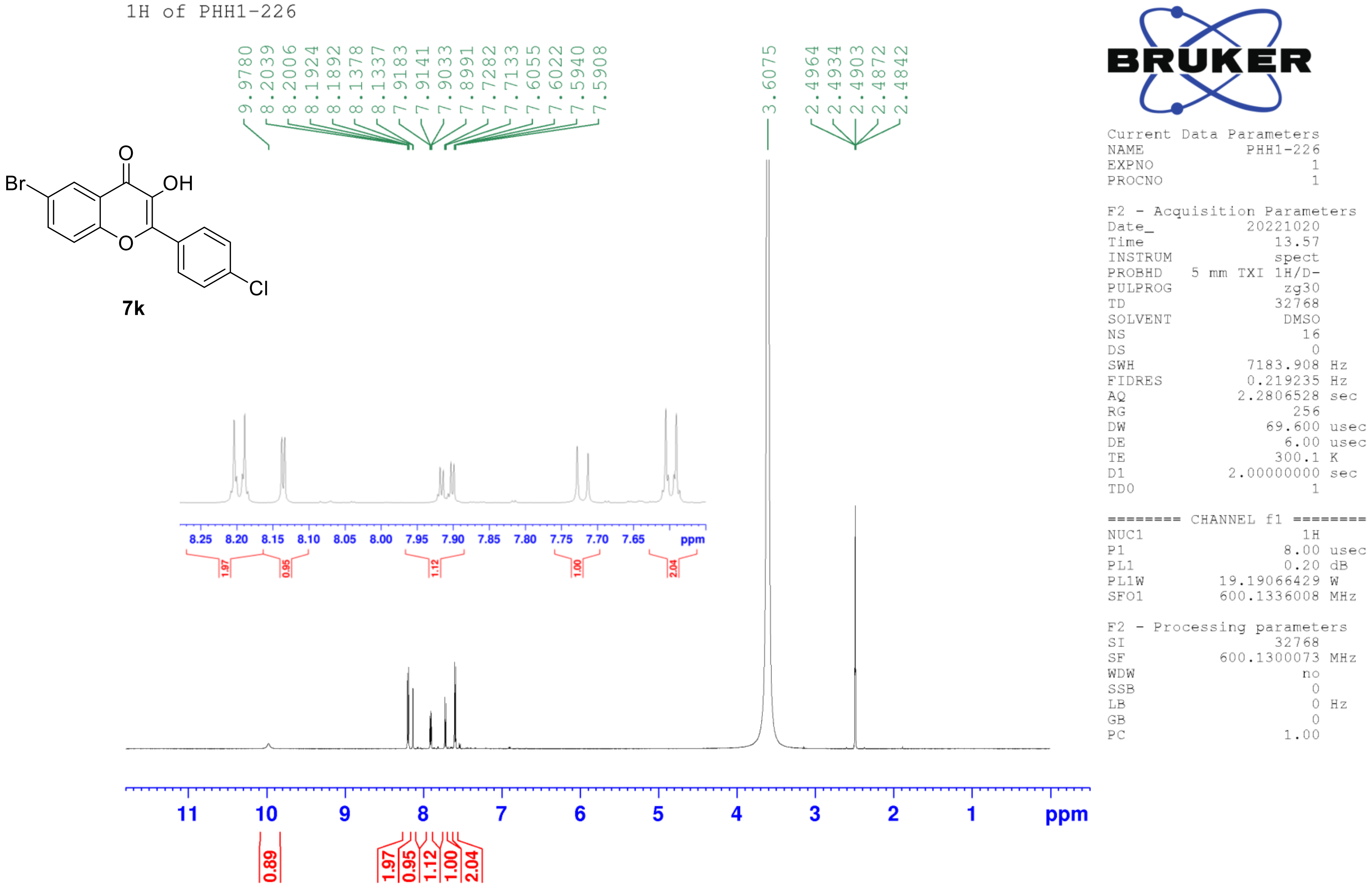

Figure S29. ^{1}H NMR (600 MHz, DMSO-d_6) for compound **7k**.

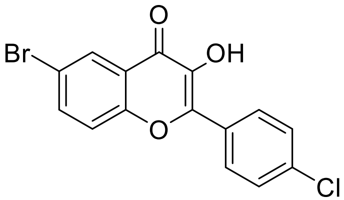

7k

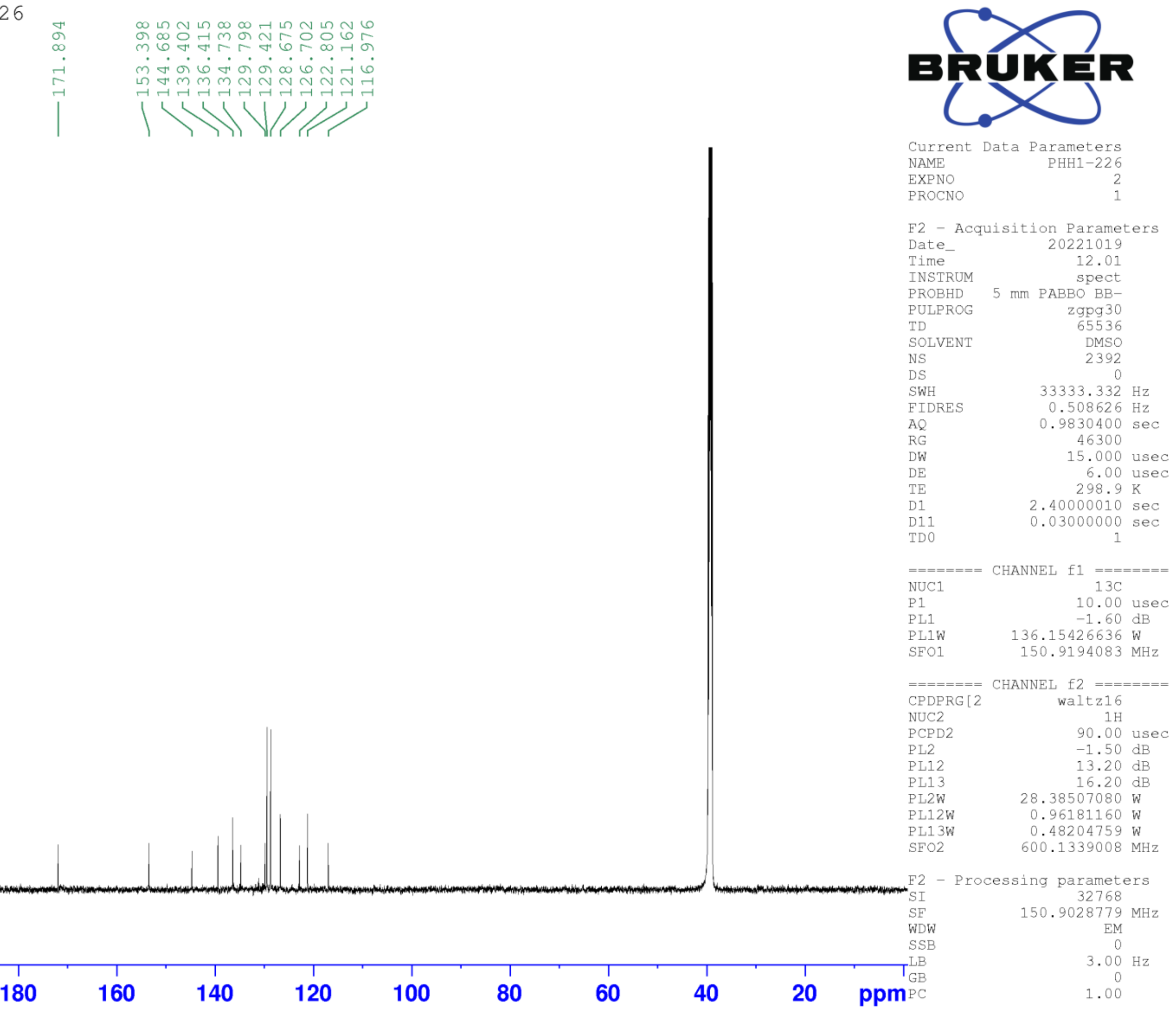

Figure S30. ^{13}C NMR (150 MHz, DMSO-d_6) for compound **7k**.

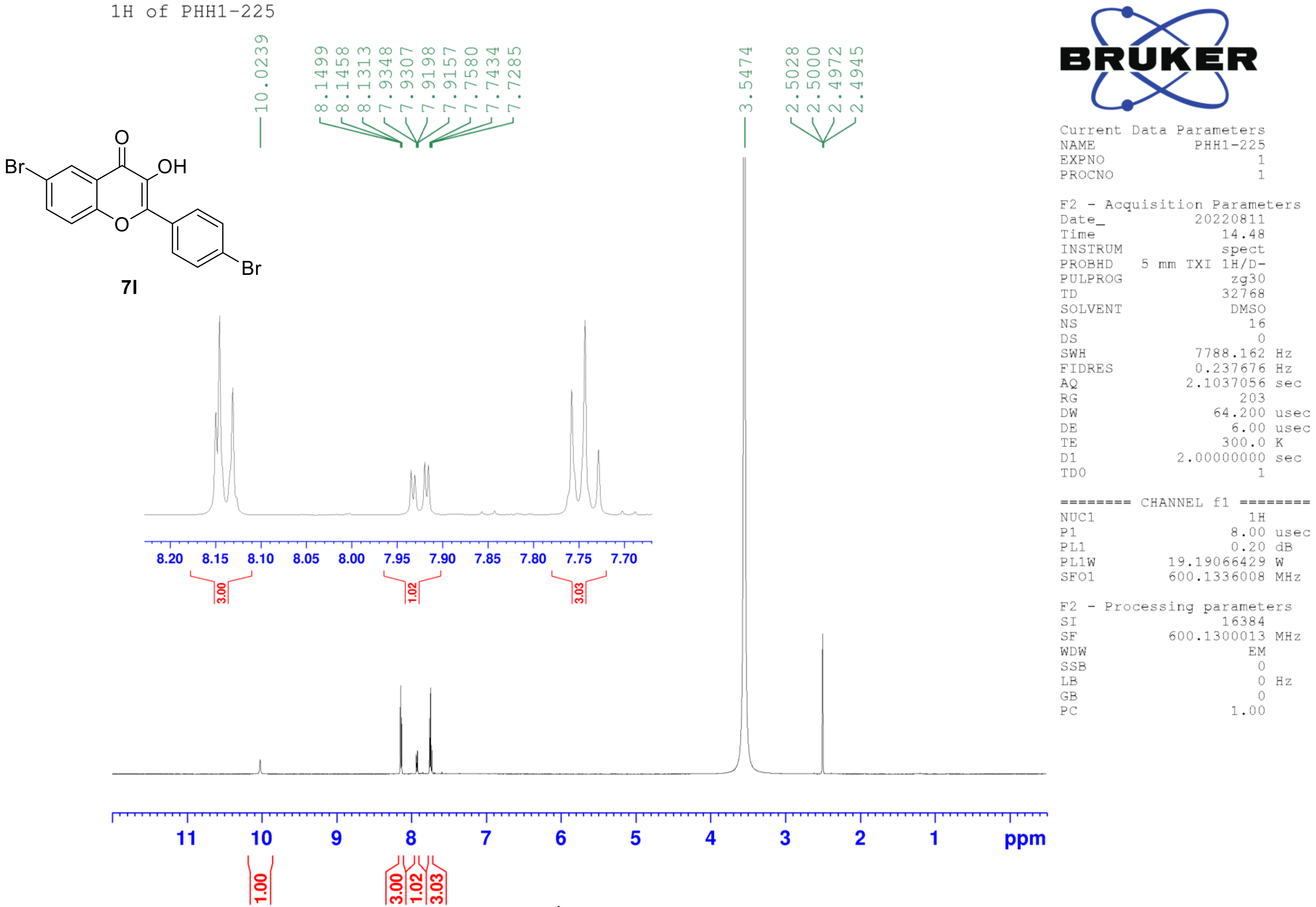

Figure S31. ^{1}H NMR (600 MHz, DMSO-d_6) for compound **7l**.

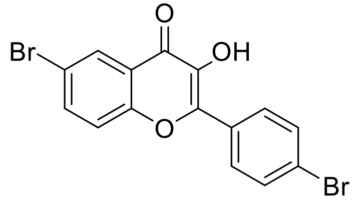

7l

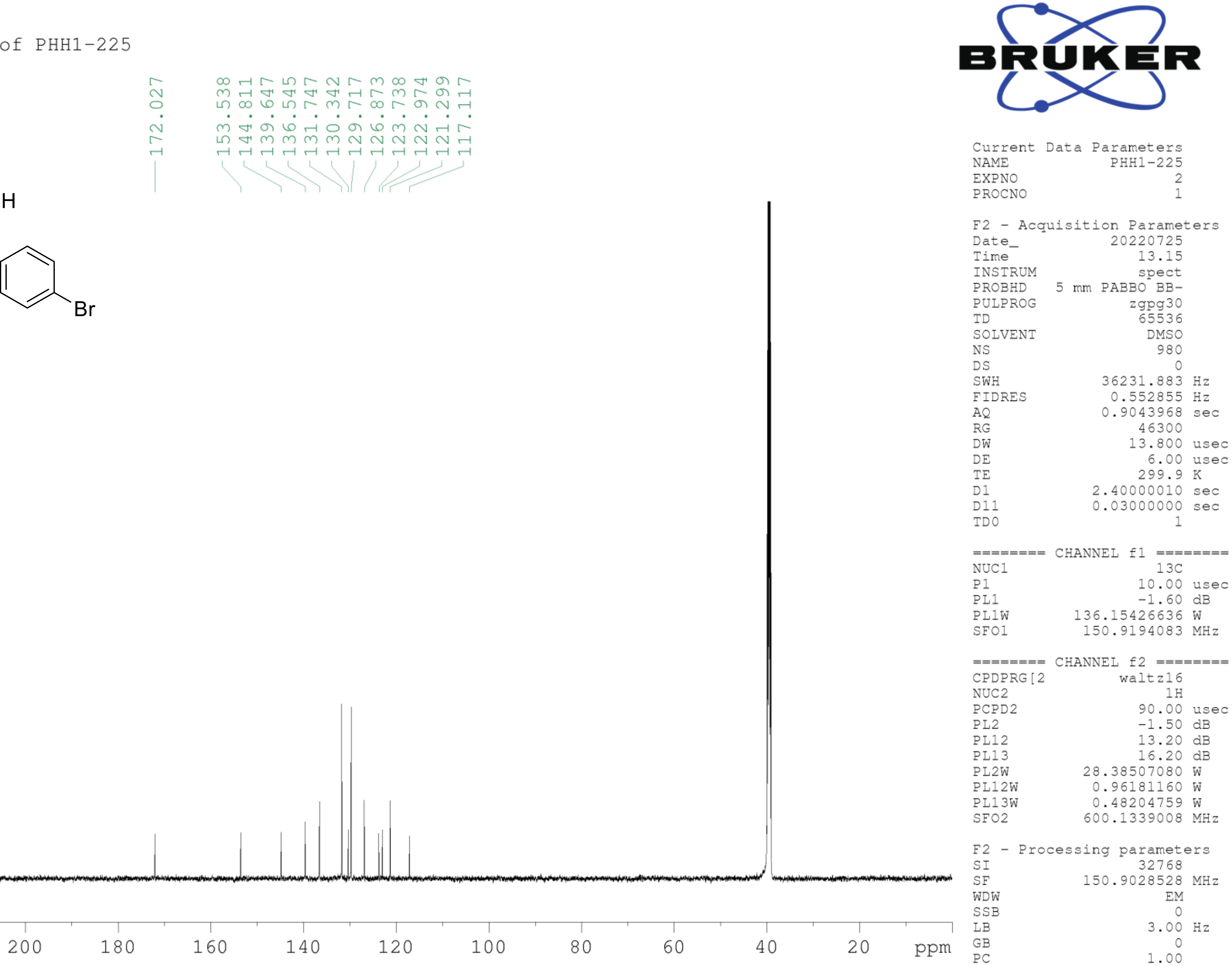

Figure S32. ^{13}C NMR (150 MHz, DMSO-d_6) for compound **7l**.
